# The plant rhizosheath–root niche is an edaphic “mini-oasis” in hyperarid deserts with enhanced microbial competition

**DOI:** 10.1038/s43705-022-00130-7

**Published:** 2022-06-03

**Authors:** Ramona Marasco, Marco Fusi, Jean-Baptiste Ramond, Marc W. Van Goethem, Kholoud Seferji, Gillian Maggs-Kölling, Don A. Cowan, Daniele Daffonchio

**Affiliations:** 1 King Abdullah University of Science and Technology (KAUST), Biological and Environmental Sciences and Engineering Division (BESE), Thuwal, 23955-6900 Saudi Arabia; 2 Centre for Microbial Ecology and Genomics, Department of Biochemistry, Genetics and Microbiology, University of Pretoria, Pretoria, South Africa; 3 Department of Molecular Genetics and Microbiology, Pontificia Universidad Católica de Chile, Santiago, Chile; 4 Gobabeb—Namib Research Institute, Walvis Bay, Namibia

**Keywords:** Microbial ecology, Environmental microbiology

## Abstract

Plants have evolved unique morphological and developmental adaptations to cope with the abiotic stresses imposed by (hyper)arid environments. Such adaptations include the formation of rhizosheath–root system in which mutualistic plant–soil microbiome associations are established: the plant provides a nutrient-rich and shielded environment to microorganisms, which in return improve plant-fitness through plant growth promoting services. We hypothesized that the rhizosheath–root systems represent refuge niches and resource islands for the desert edaphic microbial communities. As a corollary, we posited that microorganisms compete intensively to colonize such “oasis” and only those beneficial microorganisms improving host fitness are preferentially selected by plant. Our results show that the belowground rhizosheath–root micro-environment is largely more hospitable than the surrounding gravel plain soil with higher nutrient and humidity contents, and cooler temperatures. By combining metabarcoding and shotgun metagenomics, we demonstrated that edaphic microbial biomass and community stability increased from the non-vegetated soils to the rhizosheath–root system. Concomitantly, non-vegetated soil communities favored autotrophy lifestyle while those associated with the plant niches were mainly heterotrophs and enriched in microbial plant growth promoting capacities. An intense inter-taxon microbial competition is involved in the colonization and homeostasis of the rhizosheath zone, as documented by significant enrichment of antibiotic resistance genes and CRISPR-Cas motifs. Altogether, our results demonstrate that rhizosheath–root systems are “edaphic mini-oases” and microbial diversity hotspots in hyperarid deserts. However, to colonize such refuge niches, the desert soil microorganisms compete intensively and are therefore prepared to outcompete potential rivals.

## Introduction

Deserts are heterogeneous habitats that cover approximately a third of the global land surface [1] and which are expanding with global climate change [2, 3]. In addition to aridity, numerous abiotic stresses are imposed on hot desert indigenous (micro)biota, including oligotrophy, elevated daily temperatures and radiation, high salinity, eolian erosion and environmental-physical instability [1, 4, 5]. Consequently, deserts are characterized by a lower biodiversity than more temperate ecosystems [3, 6], and are populated by macro- and microorganisms adapted to poly-extreme conditions [7–9].

Xerophytic plants are desert specialists that play key roles in desert ecosystem functioning. These plants have evolved both the aerial (stem and leaf) and subterranean (root system) organs to prevent water loss, improve water storage and optimize water and nutrient uptake [4, 10–12]. Thus, xerophytic plants represent ecological and fertility islands [13] where animals and microorganisms can find shelter, nutrients and thermal protection [10, 14]. Among these, desert speargrass species of the *Poaceae* family represent highly successful examples found in most deserts [4, 15]. The majority of speargrasses growing in arid soils develop a rhizosheath (RS)–root system constituted by soil particles (sand and other small mineral particles) that physically adhere to the surface of the entire root system [15–18]. Root hairs, fungal hyphae and adhesive agents, such as microbial- and plant-derived mucilages, are responsible for the aggregation of soil particles along the RS structure and for water/nutrient retention and uptake [17, 19–21]. Therefore, RS–root systems ultimately improve the overall fitness of plants under stressful environmental conditions [22–24].

The higher moisture and nutrient contents of RS (compared to non-vegetated (NV) soil) create favorable niches for colonization by edaphic microorganisms [15, 18, 25, 26]. In return, the RS microbiome provides beneficial plant growth promoting (PGP) services by improving the plant’s nutrient (e.g., P, N and Fe) status, growth, and/or resistance to abiotic and biotic stresses, such as drought, salinity and phytopathogens [27–32].

Inevitably, the process of colonizing and subsequently residing in the favorable RS niche must involve complex microbe–microbe competition dynamics [33, 34]. By using amplicon and shotgun metagenomic sequencing, we aimed to evaluate the microbial competition–colonization course associated with the RS–root system in xerophytic desert plants. We used the perennial *Stipagrostis ciliata* (Desf.) De Winter var. *capensis* (Trin. & Rupr.) De Winter (*Poaceae* family)—locally known as “Tall bushman-grass”—as the model plant in the study. This species grows extensively in the Namib Desert gravel plains [35, 36] and has shown the capacity to rapidly establish RS structure and biotic interactions with the surrounding soil microbiota after limited rain events (10–20 mm precipitation [35]). We hypothesize that (1) the compartmentalization of the RS–root structures of *S. ciliata* (root tissues (RT), RS, rhizosphere (RH)) will lead to deterministic processes dominating the assembly of its associated microbial communities [15] and that (2) the communities from the less extreme niches (the cooler and more humid RS and RH) would be enriched in microbial cells and stable than those from the most extreme NV soils [15, 37]. Furthermore, by adapting the Darwinian “Survival of the fittest” theory [38] to the microbial world, we expect that (3) desert soil microorganisms strongly compete to colonize the protected favorable RS–root system niches. Consequently, we anticipate that the microbial communities colonizing this niche will present significantly more markers indicative of competitive interactions (e.g., antibiotic production and resistance) to remain in the refuge than those inhabiting the NV soil. Similarly, we predict that (4) the microbial communities associated with plant will exhibit significantly more PGP traits (e.g., biopromotion and biofertilization) to favor the host and their own survival in such extreme environment.

## Material and methods

### Rhizosheath–root system sampling and processing

**Fig. 1:**
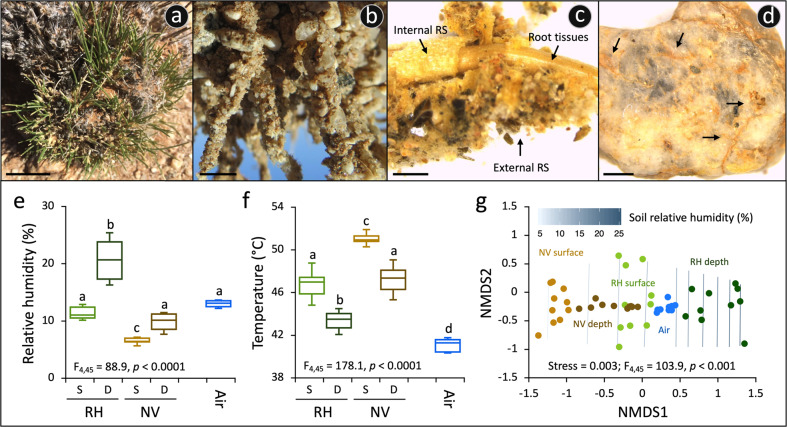
Rhizosheath–root system niche of *S. ciliata* in Namib Desert gravel plain. **a** The speargrass *S. ciliata* growing in the gravel plain as shown by the new green leaves developing from the basal portion of the plant (bar, 2 cm). **b** Close-up photograph of the *S. ciliata* rhizosheath–root system extracted from the soil (bar, 1 cm). The rhizosheath (RS) is composed of sand grains physically attached to the root, along with trapped stones and sand grains. **c** Stereomicroscope image of the rhizosheath–root system structure shows the external RS layer of the matrix with long root hairs developing from the epidermis (internal layer of RS; i.e., outermost cells of the root) that entrap sand grains and stones, as well as the central root tissue of vascular plants (bar, 1 mm). **d** Magnification of a stone detached from the RS; biological mineral weathering is indicated by black arrows (bar, 1 mm). **e** Relative humidity (%) and **f** temperature (°C) measured (*n* = 10; ±standard deviation) in NV soils (surface and in-depth) and soils under *S. ciliata* plants (surface and in-depth RH); S, surface, and D, in-depth. Values from air are also reported. Results of the ANOVA main test are indicated, along with lowercase letters referring to results of the post-hoc multiple comparison Tukey’s tests. **g** Non-parametric multidimensional scaling (NMDS) ordination plot showing the relative distribution of humidity and temperature measured; the relative humidity trend is plotted onto the ordination space. The result of the PERMANOVA main test is reported.

To cope with the hyperarid and oligotrophic conditions of the desert habitat, *S. ciliata* rapidly responds to rare and limited moisture events, adopting an amphiphytic lifestyle; i.e., either facultatively annual from seed growth or perennial by re-sprouting from existing live grass clumps [35]. In April 2017, in an area of 1 km^2^ of the Namib Desert gravel plains (S 23°32’53”; E 15°08’11”; Supplementary Fig. S1a), a total of ten *S. ciliata* plants of similar size were randomly selected to collect the RS–root systems. All selected plants had green leaves growing from the base of the clump, as evidence of active growth (Fig. 1a and Supplementary Fig. S1b, c). The entire plants were carefully exhumed from the rocky soil to preserve their RS–root systems (Fig. 1b). Intact portions of the RS–root system were subsequently excised from each plant using sterile scissors and tweezers and transferred into sterile plastic tubes (example of excised RS portion in Supplementary Fig. S1d). In addition, control NV soil samples were collected (0–10 cm depth; *n* = 10) at approximately one meter from each plant. All samples were collected under the research/collecting permit number 2248/2017, issued by the Namibian Ministry of Environment and Tourism.

Soil and air temperature and relative humidity were measured in situ using handheld Ebro Electronic™ Hygrothermometer (TFH 620 with TPH 100 Air Probe) during five consecutive days (April 24th–28th; *n* = 10). Measurements were conducted between 12:00 and 13:00 p.m. as these were the hottest hours of the day in the central Namib Desert gravel plains [39]. The edaphic measurements were recorded at the soil surface (first 5 cm) and shallow sub-surface (from 5 to 10 cm deep) from two contrasting niches: NV soils and within *S. ciliata* plant clumps in the vicinity of their RS–root system. Data were analyzed by performing non-metric multidimensional scaling on the Euclidean distance matrix in R [40, 41]; permutational multivariate analysis of variance (PERMANOVA) was performed in Primer v.6.1 [42] using sample categories as explanatory variables (five levels: under plant sub-surface or surface, NV soil sub-surface or surface, and air).

In the laboratory, portions of the RS–root system were visualized with a stereomicroscope (Leica S8 APO) to define and divide the different compartments (i.e., RT, RS and RH) and measure the average sizes of RT and RS from a portion of RS–root from each plant. The RH was characterized as loosely adherent sand, detachable from the RS by gentle shaking. The RS (root coating containing sand and mineral particles physically trapped by the root hairs along the entire length) and RT (internal tissues) were separated with sterile scalpels [15].

The NV soil (*n* = 3), RH (*n* = 3) and RS (*n* = 3) samples were used for physicochemical analyses (2–3 g per sample); water content, pH, conductivity, salinity, organic matter (OM), carbon (TC, POC, PIC) nitrogen (TN, PON, PIN, nitrite, nitrate and ammonium), phosphate (PO_4_^3−^), silicate, potassium (K), calcium (Ca), magnesium (Mg), and sulfur (S) contents were analyzed by the commercial company GEOMAR (Wischhofstraße, Germany) using standard protocols. Data were analyzed by performing PERMANOVA in Primer v.6.1 [42] on the Euclidean distance matrix, using sample categories as explanatory variables (three levels: NV soil, RH and RS). NV soil structure (percentages of rock, sand, silt, and clay) was also characterized at the Soil Science Laboratory of the University of Pretoria (South Africa).

### DNA extraction

The surfaces of RT obtained from the RS–root system were sterilized as previously described [43]. Briefly, the RT were soaked in 70% ethanol for 3 min, followed by sodium hypochlorite 2.5% for 5 min and 70% ethanol for 30 s. Several washes with sterile water were performed to remove any trace of the chemicals used for surface sterilization. Subsequently, all samples (sterile RT, RS, RH and NV soil) were separately homogenized in liquid nitrogen with sterile mortars and pestles. Total DNA from the homogenized edaphic samples (NV soil, RH and RS) was extracted using 0.7 ± 0.05 g of sample and the PowerSoil^®^ DNA Isolation Kit (MoBio Inc., USA), while from RT total DNA was extracted from 1 ± 0.1 g of sample and the DNeasy Plant Maxi Kit (Qiagen, Germany). The total DNA extracted was quantified on a Qubit Fluorometer using the high sensitivity dsDNA assay kit. The concentration of total DNA (ng/g of soil) extracted from RH and NV soils was further used as biomass proxies [44] and *t*-test was used to evaluate differences among the two compartments. Total DNA concentrations of the RT and of the RS compartments were not included in this comparison as large amounts of plant DNA were co-extracted.

### Amplicon library preparation for phylogenetic analysis of the prokaryotic 16S rRNA genes and microeukaryotic ITS regions

The V3–V4 hypervariable regions of the prokaryotic 16S rRNA gene were amplified using the universal primers 341f and 785f, and the microeukaryotic ITS2 region amplified using the primers ITS3f and ITS4r [15]. PCR reactions mixture of 30 μl were performed for each sample using 1 U of Platinum^®^ Taq DNA Polymerase, High Fidelity (Invitrogen) with 1× High Fidelity Buffer, 1.5 mM of MgSO_4_, 0.3 mM dNTPs mix, 0.3 µM each of forward and reverse primers, and ca. 10 ng of template DNA. The reaction conditions were as follows: denaturation at 95 °C for 5 min, followed by 25 cycles of denaturation at 95 °C for 30 s, annealing at 55 °C for 30 s, and extension at 68 °C for 45 s, final extension at 68 °C for 5 min. Total DNA extracted from sterile water and PCR mix (reagent without DNA) was used as an additional control in the amplicon PCRs; no amplification was detected by running the PCR product on 1% agarose gel. All the amplicon products obtained were used to incorporate the sequencing adapters by using the 96 Nextera XT Index Kit (Illumina). All tagged samples were pooled together, concentrated in a CentriVap DNA Concentrator (Labconco) and sequenced with the Illumina MiSeq platform at the Bioscience Core Lab, King Abdullah University of Science and Technology (Saudi Arabia). Raw forward and reverse reads for each sample were assembled into paired-end reads (minimum overlap of 30 nucleotides and maximum of one mismatch within the region) using the fastq-join algorithm (https://code.google.com/p/ea-utils/wiki/FastqJoin) and analyzed using the Qiime 1.9 pipeline. After quality filtering, trimming, dereplication, and paired-end merging of the sequences, a total of 1,830,127 (average length of 405 bases) and 3,669,396 (average length of 310 bases) sequences were obtained for prokaryotic and microeukaryotic components, respectively. Operational taxonomic units (OTUs) were clustered at 97% sequence similarity. Prokaryotic representative sequences of each OTU_97_ were searched against the SILVA 138 database [45], using *uclust* command, while microeukaryotes OTU_97_ representative were searched against the UNITE database [46], using the *blast* command. OTUs not identified as prokaryotes (i.e., chloroplast, mitochondria and unclassified or microeukaryotes (i.e., non-fungi and unclassified), and OTUs present in PCR and DNA blank controls were removed from the dataset (number of reads and OTUs removed per compartment are reported in Supplementary Table S2). OTUs showing low relative abundances (<0.01% for the prokaryotic 16S rRNA gene and <0.001% for the microeukaryotic ITS region datasets) were also removed (Supplementary Table S2). Rarefaction curves are shown in Supplementary Fig. S2.

### Alpha- and beta-diversity, taxonomic distribution, and statistical analyses

Compositional similarity matrices (Bray–Curtis (BC) of the log-transformed OTU tables) were calculated in PRIMER v.6.1 [42, 47, 48] and homogeneity of multivariate dispersions (PERMDISP) tested to evaluate the dispersion of samples for each compartment. The specific roles of RS–root system compartmentalization in explaining the variation of microbial communities were quantified using the PERMANOVA function *adonis2* in the *vegan* package in R [49]. Principal coordinates analysis (PCoA) and multivariate generalized linear models (many GLM, main and multiple comparison tests, using negative binomial family errors; Supplementary Fig. S3) were performed, with the factor “compartment” (of four levels: RT, RS, RH, and NV soil) as explanatory variables and by using PRIMER v.6.1 and the R package *mvabund* [50], respectively. Components of beta-diversity (similarity, richness difference and replacement) in the microbial communities associated with the root-system compartments and the NV soil were also quantified using the function *beta.div.comp* of the R package *adespatial* [51, 52]. The occurrence of distance-decay patterns in the RS–root system and NV soil was tested using the linear regression (GraphPad Prism 7 software, La Jolla California, USA) between the similarity of bacterial/microeukaryotic communities (BC) and the distance between the different compartments. Alpha diversity indices (richness and evenness) were calculated using the PAST software [53]. Shared and exclusive bacterial and microeukaryotic OTUs (fungi and algae) in the different compartments were visualized using Venn-diagrams. The Kruskal–Wallis test (FDR *p* correction) was used to detect significant differences (*p* < 0.05) among taxonomic groups in RS–root system compartments.

### Co-occurrence network analysis

To identify ecological clusters of strongly associated microorganisms, a co-occurrence network was generated for each RS–root system compartment and for the NV soils using the CoNet plugin of Cytoscape 3.4 [54, 55] and Gephi 0.9.1 [56] for computation and visualization, respectively. We used the compartment-OTU tables described above for the analysis and merged the bacterial and microeukaryotic datasets using their relative abundance. We considered all the OTUs present in a compartment (i.e., RT, RS, RH and NV soil) to identify co-occurring OTUs within each network, possibly indicating functional/physical interactions among them in the different compartments. A combination of BC and Kullback–Leiber dissimilarity indices, along with the Pearson and Spearman correlation coefficients were used to build the networks. Edge-specific permutation and bootstrap score distributions with 2000 iterations were performed. For each measure and edge, 100 permutations and bootstrap scores were generated. The obtained data were normalized to detect statistically significant non-random events of co-occurrences, i.e., co-presences and mutual exclusions. The *p* values were computed by *z*-scoring the permuted null and bootstrap confidence intervals using pooled variance [57]. The most important statistical network descriptors were calculated [58], along with the normalized degree (number of node connections standardized by the total number of connections [59]), node betweenness centrality, and frequency of edges connecting the three microbial components; i.e., bacteria–bacteria, fungi–fungi, bacteria–fungi, and algae–algae/bacteria/fungi. Hubs (nodes with degree >75th percentile among all network nodes) and keystone taxa (nodes with betweenness centrality >75th percentile among hubs) were identified in the final networks.

### Metagenome library preparation, sequencing and analyses

A total of nine samples (three RS, three RH and three NV soil) were selected for metagenomic analyses. Metagenomic libraries were prepared at the Bioscience Core Lab, King Abdullah University of Science and Technology (Saudi Arabia). First, 100 ng of total DNA were diluted in 52.5 ml of Nuclease-Free water (Ambion), in Covaris snap cap microtube (PN 520045, Covaris) and it was further fragmented by sonication with Covaris M220 to target DNA fragments of 300 bp, following this sonication protocol: treatment time 70 s, cycle per bust 200, duty factor 20%, peak incident power 50 W. The 300 bp fragmented DNA was used as input to prepare metagenomic libraries by using Illumina TruSeq Nano DNA Library Prep kit according to manufacturer’s instructions. Individual libraries were pooled, and the quality and quantity checked using a BioAnalyzer (Agilent) and the KAPA Library Quantification Kit (Roche). Pooled libraries were loaded on Illumina NovaSeq 6000 using 150 bp × 2 paired-end sequencing with an S1 flow cell following the NovaSeq XP workflow at the Biological Core Lab, King Abdullah University for Science and Technology (Saudi Arabia). Raw read sequences were quality filtered and trimmed using Trimmomatic v0.32 [60] to remove adapter sequences and leading and trailing bases with a quality score below 20 and reads with an average per base quality of 20 over a 4-bp window. The metagenomes were quality checked and filtered using FastQC v0.11.9 [61] and PRINSEQ-lite v0.20.4 to remove short sequences and those with ambiguous bases [62]. The sequencing depth of each metagenome was assessed using Nonpareil 3 [63]. All metagenomic reads were taxonomically classified using Kraken 2 [64] against the standard NCBI RefSeq database (accessed April 2022). Kraken 2 uses a k-mer based lowest common ancestor method with whole genome references to estimate read taxonomy to the deepest possible taxonomic resolution. High-quality reads were then assembled into contigs using metaSPAdes v3.12.0 [65]. The assembled metagenomes were quality checked using MetaQUAST v5.0.2 [66] after which we identified open reading (ORFs) using Prodigal v2.6.3 [67] through Prokka v1.12 [68]. These data were searched for genes related to plant growth-promotion (PGP, i.e., biofertilisation, biopromotion and bioprotection), nutrient and energy acquisition (N and P cycling, cellular metabolism, and energy acquisition) and competition (biotic competition and trophic interaction); the list of genes analyzed is given in Supplementary Table S3. Ribosomal genes were identified and taxonomically annotated from the unassembled sequence data using SingleM from which an OTU table of unique sequences was constructed. Secondary metabolic biosynthetic gene clusters (BGCs) were identified from contigs longer than 5 kb using antiSMASH v5.0 [69] under strict settings which only detects well-defined clusters containing all required parts. Carbohydrate-active enzymes were analyzed against the dbCAN2 database [70] whereby hits in two of the following databases were required to be accepted: HMMER (*E* value <1e^−15^, coverage >0.35), DIAMOND [71] (*E* value <1e^−102^) and Hotpep (Frequency > 2.6, Hits >6). Using the standards reported in Supplementary Methods S1, metagenome-assembled genomes (MAGs) were assembled with high-quality (>90% complete, <5% contamination) and medium-quality (>50% complete, <10% contamination) drafts.

## Results

### Rhizosheath–root systems of *S. ciliata* create favorable resource islands

The NV soils of the central Namib Desert gravel plain were extremely oligotrophic (0.33 ± 0.01 µg/mg of organic carbon (POC) and 0.046 ± 0.01 µg/mg of total nitrogen (TN)) and were structurally constituted by fine/very-fine sand particles and stones (Table 1 and Supplementary Table S4). In these soils, *S. cilata* is the dominant plant species [35] and often constitutes a monospecific vegetation cover after rain events exceeding 20 mm (Supplementary Fig. S1a). All the *S. ciliata* plant clumps tested (Fig. 1a and Supplementary Fig. S1b, c) exhibited a well-developed RS–root system (Fig. 1b). The RS matrix (RS and RH) appeared as thick and compact sandy cylinder, covering the entire length of each root (Fig. 1b, c and Supplementary Fig. S1d) and showed significantly different physicochemical properties from the surrounding NV soils (PERMANOVA: *F*_2,6_ = 37.53, *p* = 0.001; pairwise comparison of RS/RH vs. NV soil: *t* = 8.45, *p* = 0.001 and *t* = 2.61, *p* = 0.017, respectively). In general, RH and RS were less oligotrophic than NV soils with significantly higher concentrations of total carbon (TC; organic [OM, POC]) and total nitrogen (TN; organic [PON] and ammonium [NH_4_^+^]; Table 1 and Supplementary Table S4); the inorganic C/N forms [PIC and PIN] were only enriched in the RH. The RS soils had also the highest concentrations of phosphate [PO_4_^3−^], calcium [Ca], magnesium [Mg] and sulfur [S] (Supplementary Table S4). Notably, the rocks associated with the RS showed signs of biological minerals weathering, possibly mediated by microorganisms and *S. ciliata* roots and root hairs (Fig. 1d and Supplementary Fig. S1e–g).

**Table 1:** Characterization of carbon and nitrogen contents of the gravel plain non-vegetated (NV) soil, speargrasses rhizosheath (RS) and rhizosphere (RH); values are expressed as mean of three replicates ± standard deviation.

Variable	Unit	ANOVA (*p*)	NV	RH	RS
Organic matter	%	0.0008	0.066 ± 0.002 (a)	2.869 ± 0.723 (b)	0.140 ± 0.076 (a)
Total C	µg/mg	<0.0001	2.030 ± 0429 (a)	17.185 ± 2.962 (b)	2.199 ± 0.886 (a)
POC	µg/mg	0.0008	0.332 ± 0.010 (a)	14.344 ± 3.616 (b)	0.698 ± 0.382 (a)
PIC	µg/mg	0.0197	1.698 ± 0.421 (a)	2.842 ± 2.130 (b)	1.502 ± 0.622 (ab)
Total N	µg/mg	0.0021	0.038 ± 0.100 (a)	0.418 ± 0.100 (b)	0.056 ± 0.021 (a)
PON	µg/mg	0.0074	0.043 ± 0.002 (a)	0.357 ± 0.099 (b)	0.056 ± 0.018 (a)
PIN	µg/mg	<0.0001	0.005 ± 0.004 (a)	0.0611 ± 0.008 (b)	0.0006 ± 0.002 (a)

Results of ANOVA tests are reported (*p* value); different letters indicate the significant difference across samples (Tukey’s multiple comparisons test, *p* < 0.05). The other physicochemical parameters measured are listed in Supplementary Table S4.

*POC* particulate organic carbon, *PIC* particulate inorganic carbon, *PON* particulate organic nitrogen, *PIN* particulate inorganic nitrogen, *NV* non vegetated, *RH* rhizosphere, *RS* rhizosheath.

The relative humidity of surface and sub-surface NV soils was significantly lower than the soils under the plants (RH, Fig. 1e). The niche that showed the highest relative humidity values was the sub-surface RH (ranging from 17 to 25%), while those with the lowest values was the surface of NV soil (6% ± 0.5%). Both these variables varied significantly with the distance between *S. ciliata* individuals and the corresponding NV soils (relative humidity: *p* < 0.0001, *R*^2^ = 0.81, *r* = −0.89; temperature: *p* < 0.0001, *R*^2^ = 0.78, *r* = 0.88). Consequently, day ambient temperatures, which ranged from 40 °C to 52 °C, were significantly lower in the RH of speargrass clumps than in NV soils at equivalent depths (4.2 °C ± 1.3 °C and 3.8 °C ± 1.2 °C cooler for surface and sub-surface horizons, respectively; Fig. 1f). The field parameters measured showed the presence of an environmental gradient, with the more favorable conditions in the deep *S. ciliata* RH and the harshest conditions in the surface NV soils (Fig. 1g). Altogether, these results and the fact that the most recent rain event recorded occurred over a month prior to sampling (Supplementary Table S1) show the enhanced water retention capacity of the RS–root systems.

### Niche-partitioning dominates the assembly of *S. ciliata* rhizosheath–root microbial communities’ structure

**Fig. 2:**
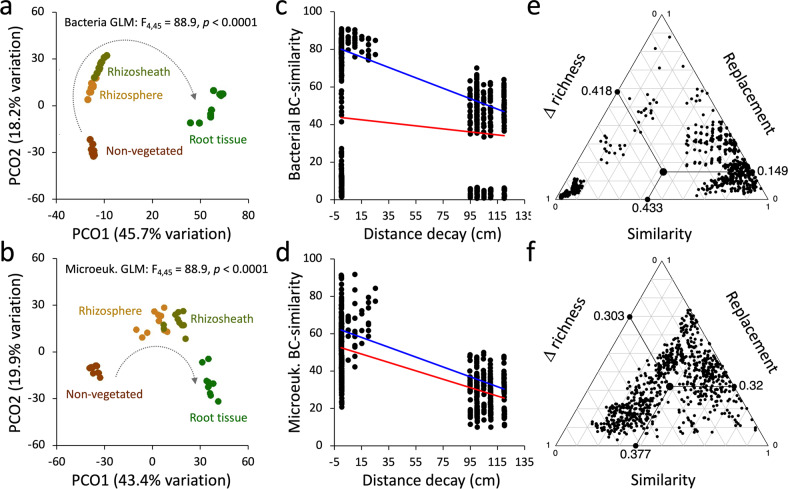
Diversity and dynamics of microbial communities associated with the *S. ciliata* rhizosheath–root system. Principal coordinate analysis (PCoA) of (**a**) bacterial and (**b**) microeukaryotic communities associated with the rhizosheath–root system (root tissue (RT), rhizosheath (RS) and rhizosphere (RH)) and non-vegetated (NV) soil. Arrows indicate the “horseshoes” shape distribution of microbial communities associated to different compartments, starting from the RT, and ending into the NV soils. Decay relationships among microbial communities’ similarities (BC: Bray–Curtis) and compartment relative distance (cm) for (**c**) bacteria and (**d**) microeukaryotes; red regression lines indicate the significant correlation among BC’ similarity and distance considering all the compartments. We note that when excluding the root tissues from the analyses, the correlation coefficients increased; blue regression lines, bacteria: *p* < 0.0001, *R*^2^ = 0.81; microeukaryotes: *p* < 0.0001, *R*^2^ = 0.73. Ternary plot presenting the variation in species composition among sites (beta-diversity) as result of its three components: similarity, replacement and richness difference [51]; the indices decomposing beta-diversity are visualized for the (**e**) bacterial and (**f**) microeukaryotic communities. Each point represents a pair of samples, and its position is determined by a triplet of values from the similarity, replacement, and richness difference. In each ternary plot, the large central dots where the lines start are the centroid of the points for each beta-diversity component; the lines represent their mean values.

The “horseshoe shape” distribution of samples in the PCoA ordination plots reveals that both bacterial and microeukaryotic communities were assembled via niche-partitioning processes along the RS–root system compartments (manyGLM, *F*_3,36_ = 64815, *p* = 0.001 and *F*_3,36_ = 7196, *p* = 0.001, respectively; Fig. 2a, b and Supplementary Tables S5 and S6). Even though the NV soils was the microbial-source of the vast majority of the plant-associated bacterial and microeukaryotic OTUs (91% of OTUs (96% of relative abundance) and 56% of OTUs (97% of relative abundance), respectively; Supplementary Fig. S5), the selective process exerted by the RS–root system defined a non-random distribution of OTUs across the compartments with a significant relationship between microbial occurrence and abundance (Supplementary Fig. S6). The niche partitioning was further confirmed by the significant decline in compositional BC similarities with increasing distance; i.e., the edaphic microbial communities associated with close compartments (RH and RS) were more similar than those from the distant NV soils for both bacteria and microeukaryotes (*p* = 0.0003, *R*^2^ = 0.02 and *p* < 0.0001, *R*^2^ = 0.45; red regression lines in Fig. 2c, d). While the dissimilarities among bacterial communities were determined by similarity and richness differences, for microeukaryotes it was equally driven by the three components (i.e., similarity, richness differences and replacement; Fig. 2e, f and Supplementary Result S1). Despite such difference, the significant and positive relationship between bacterial and microeukaryotic BC similarity matrices (*F*_1,778_ = 523.9, *p* < 0.001, *R*^2^ = 0.4; considering only edaphic compartments: *F*_1,433_ = 1464, *p* < 0.001, *R*^2^ = 0.77) indicated commonalities in the processes driving the assembly, diversity and composition of the two microbial communities’ components at the RS–root system micro-scale.

The impact of niche partitioning was also reflected in the microbial community compositions of each compartment. Significant compartment-specific differences for bacterial and microeukaryotic richness (*F*_3,36_ = 834.3, *p* < 0.0001 and *F*_3,36_ = 41.8, *p* < 0.0001, respectively) and evenness (*F*_3,36_ = 126, *p* < 0.0001 and *F*_3,36_ = 10.4, *p* < 0.0001, respectively) were detected. Specifically, lower species richness and evenness metrics were detected in RT and increased outwards from the plant in the RS up to reach the highest values in the RH and NV soils (Supplementary Table S7). However, the concentrations of the total DNA extracted, which can be used as a proxy for microbial biomass in desert soils [44], showed significantly higher values in the RH than in the NV soils (357 ± 137 vs. 85 ± 33 ng DNA/g soil, respectively; *t*_1,18_ = 6.097, *p* < 0.0001), revealing a local enrichment of microbial cells in the vicinity of the plant.

**Fig. 3:**
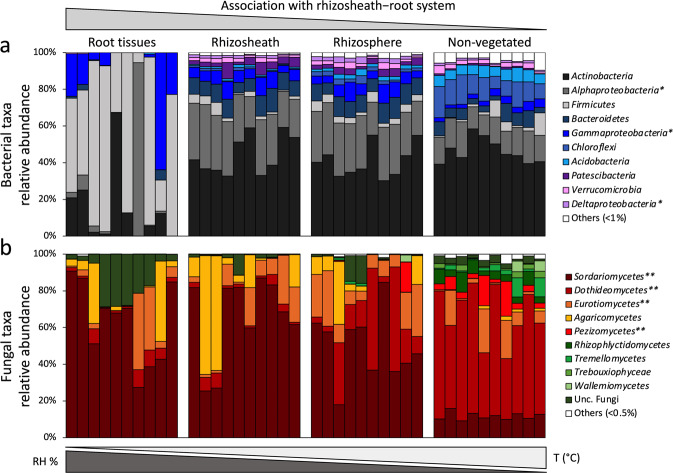
Taxonomical composition of microbial communities associated with the *S. ciliata* rhizosheath–root system and non-vegetated soil. Relative abundance of (**a**) bacterial and (**b**) microeukaryotic phyla/classes associated with rhizosheath–root system compartments (root tissue (RT), rhizosheath (RS) and rhizosphere (RH)) and non-vegetated (NV) soil. Relative abundances are expressed as percentages; star (*) indicates classes belonging to the *Proteobacteria* phylum; stars (**) indicate classes belonging to the *Ascomycota* phylum. Gradients of relative humidity and temperature are also schematically reported on the top.

The edaphic bacterial communities were consistently dominated by *Actinobacteria* (45%, 41% and 47% of the RS, RH and NV soil communities, respectively), *Alphaproteobacteria* (27, 26 and 15%) and *Bacteroidetes* (9, 9 and 6%), while RT bacterial communities were dominated by *Firmicutes* (59%) and *Gammaproteobacteria* (14%) (Fig. 3a; Supplementary Table S8 and Supplementary Data S1). The microeukaryotic communities were less diverse; *Dothideomycetes*, *Pezizomycetes*, *Tremellomycetes*, and *Rhizophylyctidomycetes* classes dominated the NV soils, while *Sordariomycetes* and unclassified fungi were significantly enriched in all compartments of the speargrass RS–root systems (RT, RS and RH; Fig. 3b; Supplementary Table S8 and Supplementary Data S1).

### Rhizosheath–root system niches enhance microbial connectivity and network stability

**Fig. 4:**
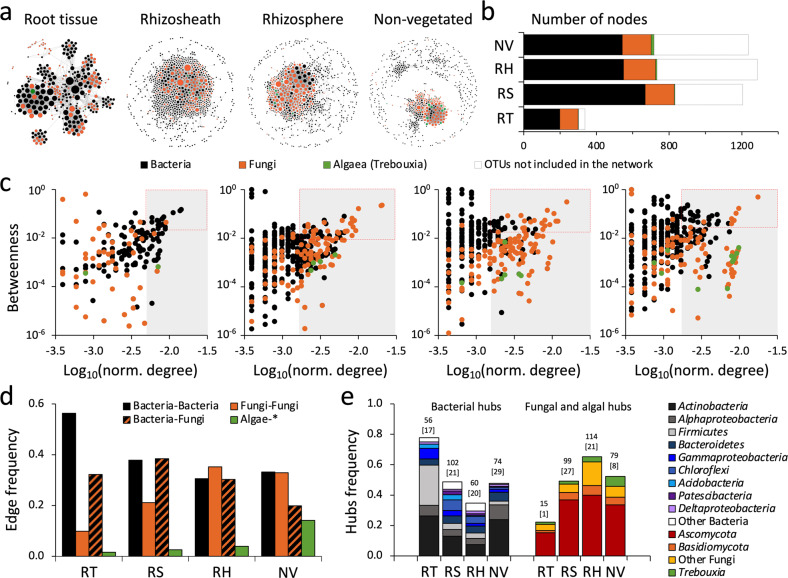
Bacterial and fungal co-occurrence network in *S. ciliata* rhizosheath–root system compartments. **a** Significant interactions (edge, *p* < 0.05) between bacterial and fungal OTUs in root tissue (RT), rhizosheath (RS) and rhizosphere (RH) of *S. ciliate* and non-vegetated (NV) soil were visualized by co-occurrence network. Circles (nodes) represent individual OTUs (bacteria, fungi, and algae); size of circles indicates the number of connections of such node (degree); nodes were colored according to their taxonomic affiliation: black, orange and green indicate bacteria, fungi and algae, respectively. For co-occurrence networks’ properties refer to Table 2. **b** Proportion of bacterial, fungal and algae OTUs included in co-occurrence networks. Portion of OTUs not included in the network is also reported (white portion of bars). **c** Relationship between node-normalized degree (log_10_) and betweenness centrality in networks of rhizosheath–root system and non-vegetated soil compartments. Colors indicate the taxonomic affiliation of nodes (bacterial, fungal and algal OTUs); gray-box indicate the nodes with high degrees defined as hubs; red-dashed boxes delineate the keystone species of each network (high degree and high betweenness). **d** Frequency of edges (connections) in the rhizosheath–root system compartments’ networks. Connections among the three components of the network are showed: bacteria–bacteria, fungi–fungi, bacteria–fungi, and algae with all the others (* = bacteria, fungi and algae). **e** Taxonomic affiliation of bacterial and fungal hubs detected for each network (**c**) expressed as normalized frequency; total number of bacterial and fungal hubs are reported on each bar, along with the number of keystone species accounted among them (numbers in square brackets). Details regarding taxonomy are reported in Supplementary Data S2.

A total of 66% (±15%) of all microbial OTUs significantly co-occurred with one another (Fig. 4a, b and Table 2). Networks based on these co-occurrences were bacteria-dominated (66–80% of nodes). Fungal OTUs represented the remaining portion (19–33% of nodes), along with algal OTUs (<2% of nodes). A reduction in heterogeneity and modularity, together with an increase in connectedness and centrality of nodes, was observed from the NV soils to the RT (Fig. 4a and Table 2). The analysis of the node connectivity (i.e., normalized degrees per node) revealed a significantly higher level of connection in the RT network when compared to the edaphic networks (RS, RH and NV; ANOVA: *F*_3,2579_ = 125.7, *p* < 0.0001; Supplementary Fig. S7a). The contribution of each microbial group was compartment dependent: in RT the bacterial nodes showed the higher level of connections, while in all the edaphic compartments those of fungi were the most connected (Fig. 4c; Supplementary Result S2 and Supplementary Fig. S7b). In the RT network, the key connectors were the *Firmicutes* (including the well-known plant-associated *Bacillus* and *Paenibacillus* genera [72]), *Actinobacteria* (*Nocardioides*, *Isoptericola* and *Streptomyces*), and *Gammaproteobacteria* (*Acinetobacter*, *Ramlibacter* and *Pseudoxanthomonas*), as well as fungal PGP taxa such as *Preussia* (*Ascomycota* [73]), *Rhodotorula* (*Basidiomycota* [74]) and *Mortierella* (*Zigomycota* [75]; (Fig. 4e; Supplementary Data S2 and Supplementary Result S2). The edaphic RS, RH and NV soil networks showed significantly more fungal hubs and keystones species than the RT (Fig. 4e). Hub fungal taxa were affiliated to *Ascomycota* (among others, *Acremonium*, *Aspergillus*, *Bipolaris*, *Chaetomium*, *Chrysosporium*, *Curvularia*, *Eurotium*, *Monosporascus*, Pseudospiromastix and *Thermomyces*) and *Basidiomycota* (*Coprinopsis*, *Filobasidium*, *Rhodotorula* and *Wallemia*; Supplementary Data S2). The edaphic bacterial hubs/keystone species mostly belonged to the *Actinobacteria* and *Alphaproteobacteria* (Fig. 4e and Supplementary Data S2). It is also noteworthy that the photoautotrophic unicellular alga *Trebouxia* was ubiquitously identified as a network hub in the edaphic compartments (Fig. 4e).

**Table 2:** Co-occurrence network topology indices reported for the three rhizosheath–root system compartments (root tissue (RT), rhizosheath (RS) and rhizosphere (RH)) and the non-vegetated (NV) soil.

Network parameter	RT	RS	RH	NV
Node	301	832	733	717
N. bacteria (%)	199 (66)	669 (80)	549 (75)	542 (76)
N. fungi (%)	100 (33)	159 (19)	176 (24)	159 (22)
N. algae (%)	2 (1)	4 (1)	8 (1)	16 (2)
Interaction	2548	2550	2320	2660
N. positive (%)	2133 (84)	1484 (58)	1568 (68)	2107 (79)
N. negative (%)	415 (16)	1066 (42)	752 (32)	553 (21)
Degree^a^	17	6	6	7
Betweenness^a^	608	2354	2116	952
Betweenness centrality^a^	0.010	0.016	0.025	0.028
Cluster coefficient	0.719	0.279	0.278	0.236
Centralization	0.188	0.118	0.089	0.069
Average path length	3.167	4.678	5.52	5.939
Average neighbors	16.93	6.129	6.33	5.419
Density	0.056	0.007	0.009	0.001
Heterogeneity	0.704	1.433	1.444	1.513
Modularity	0.137	0.199	0.322	0.647

*RT* root tissue, *RS* rhizosheath, *RH* rhizosphere, *NV* non vegetated.

^a^Median values are reported for each compartment.

### Microbial communities in the root–rhizosheath–rhizosphere continuum favor a heterotrophic lifestyle

The shotgun metagenomes from the edaphic compartments (RS, RH and NV soils) supported the taxonomic results obtained by amplicon sequencing, i.e., each compartment showed taxonomically distinct communities (PERMANOVA; *R*^2^ = 0.73, adjusted *p* < 0.005; Supplementary Fig. S8; Supplementary Results S3 and Supplementary Data S3). Evaluation of indicator genes for the potential-acquisition of carbon, nitrogen and phosphorous showed evidence of different lifestyles across the edaphic niches (PERMANOVA; *R*^2^ = 0.72, adjusted *p* < 0.003). For example, compared to NV soil metagenomes, those in RS and RH were significantly enriched in genes involved in the metabolism of simple carbon substrates, such as sugars, amino acids and organic acids, that are typically present as root exudates [18, 76], as well as in carbohydrate-active enzymes, such as glycosyl hydrolases and glycoside transferases; *p* < 0.05) and cellulases (*celE*; *p* < 0.04). Similarly, N and P microbial uptake genes suggest that the RH and RS microbial communities relied on readily assimilable and/or abundant substrates (Table 1), including ammonia and nitrate (NO_3_^–^; ammonia monooxygenase subunits (*amoABC*; *p* < 0.001) and assimilatory nitrate reductases (*nas*; *p* < 0.02)), ammonia/ammonium (NH_3_/NH_4_^+^; nitrate reductase (*nrfA*; *p* < 0.03)), inorganic phosphate (low-affinity phosphate importers, *pit* (*p* < 0.002) and glycerol-3-phosphate ABC transporters *ugpABCE* (all *p* < 0.02)) and phytic acid (a plant-derived molecule; acid phosphatases (*acpp*; *p* < 0.05) and 3-phytases *phyA* (*p* < 0.03)). In the RS we also observed significant overrepresentation of transporter genes (*hsrA*, *pbuE*, sugar efflux transporter C, *hmuU*, *gsiA*), suggesting a high capacity for metabolite uptake, such as for photosynthates released by the speargrass root system.

In contrast, the NV soil communities were significantly enriched in aerobic carbon fixation capacity (*prkB*, *rbcLS*, RuBisCO; *p* < 1.52 e^−3^), assimilatory hydrogenases ((NiFe) hydrogenases; *p* < 0.015), the Wood-Ljungdahl pathway for anaerobic carbon fixation (*acdABCSD*; *p* < 0.03) and aerobic respiration (cytochrome C oxidases; *p* < 9.38 e^−3^), indicating a prioritization of energy acquisition strategies based on gasses (i.e., CO_2_ and H_2_). A preference for autotrophic metabolism was consistent with the higher proportion of biological N fixation (*nifH*) genes (*p* < 0.0004) in the NV soil metagenomes, as well as with the higher presence of genes encoding for the acquisition of inorganic phosphate (PO_4_^3–^) directly from the environment through alkaline phosphatases (*phoA*, *p* < 0.0001), the Pho regulon (*phoBR, p* < 0.02) and the high-affinity phosphate transport system (*pstSCAB*; *p* < 0.0001). Along with these, in the NV soil microbial communities we observed a significant enrichment of genes involved in abiotic stress mitigation, including DNA repair mechanisms (*radA*, *recNO*, *mutHLS*; *p* < 0.02), breakdown of reactive oxygen species (superoxide dismutase; *p* < 0.015) and UV-damage repair (*uvrABC*; *p* < 0.03).

### Rhizosheath–root system microbiome is enriched in plant-beneficial traits

**Fig. 5:**
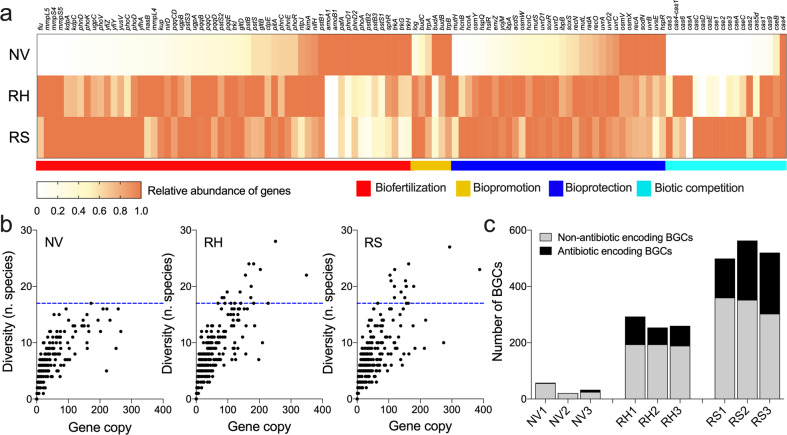
Functional potential of microbial communities from root-associated edaphic niches of the Namib Desert. **a** Heatmap showing the relative normalized abundances of metabolic marker genes from the metagenome assemblies in rhizosheath (RS), rhizosphere (RH) and non-vegetated (NV) soil. Relative abundances are calculated by first normalizing by sequencing depth and then scaling against the highest proportion for each marker gene; genes within the PGP categories biofertilization, biopromotion and bioprotection (abiotic and biotic stresses), along with genes involved in biotic competition, are used in this analysis. **b** Relationship between the copies of PGP/biotic competition genes (*x*-axis) and the number of microbial species presenting these genes (*y*-axis) is visualized in NV, RH and RS. **c** Number of biosynthetic gene clusters (BGCs) detected across RS, RH and NV soil metagenomes; antibiotic-encoding BGCs are indicate in black, while the remaining BGCs in gray.

We explored the ecological services potentially provided to the plant by the RS and RH microbiomes, focusing on three distinct plant-beneficial functions: biofertilization (solubilization of nutrients for the enhancement of plant nutrition), biopromotion (stimulation of plant growth mediated by microbial-derived phytohormones and volatile compounds), and bioprotection (mitigation of plant abiotic and biotic stresses; list provided in Supplementary Table S3). Overall, plant-associated RS and RH microbial communities were significantly enriched in PGP traits from all the three categories, compared to those associated with NV soil communities (PERMANOVA; *R*^2^ = 0.85, *p* < 0.05; Fig. 5a, b). For instance, the RS and the RH metagenomes had significantly higher abundances of biofertilization markers involved in siderophore production (catecholate siderophore receptor (*fiu*; *p* < 0.05), siderophore-binding lipoprotein (*yfiY*; *p* < 0.0001) and heme uptake protein (*mmpLS*, *p* < 0.0001)), ammonification (nitrite reductase (*nirD*, *p* = 0.002)), potassium metabolism (potassium transporters (*kimA*; *p* < 0.002, and *kdpC*; *p* < 0.0001)) and phosphate solubilization (*pqqBCDE*, *p* = 0.04) compared to the NV soil metagenomes (Fig. 5a and Supplementary Data S3). The selection of beneficial microorganisms by *S. ciliata* was also indicated by the significant enrichment in the RS and the RH metagenomes of microbial genes encoding for cytokinin (cytokinins riboside 5’-monophosphate phosphoribohydrolase (LOG9; *p* = 0.04)), ACC deaminase (*acdS*; *p* = 0.04), auxin (indole-3-glycerol phosphate synthase, *idpC*; *p* = 0.003) and exopolysaccharide synthesis (*epsF*; *p* = 0.002). Genes involved in pathogen inhibition mechanisms, such as hydrogen cyanide production (*hcnABC; p* = 0.04), chitinase B (*p* < 0.0001) and ABC transporters (*ytfQ*, *yphF*, *uup*, *ramA*, *modF*, *natB* and *natA* [77]; all *p* < 0.05) were more common in the RS and RH metagenomes than those of NV soils (Fig. 5a).

Analysis of PGP traits and taxonomic diversity revealed that the RH and RS microbial communities had a higher functional redundancy than NV soil communities (i.e., coexistence of multiple distinct taxa capable of performing a given PGP function [78]; Fig. 5b). Considering all the PGP genes analyzed, we found that RS, RH and NV had 5.6, 5.2 and 3.7 microbial species per PGP gene, respectively, and the relationship between gene copies and diversity (number of species) was significantly different among the three compartments (GLM: *χ*^2^_1,2_ = 14.35, *p* < 0.0007). RH and RS also showed a higher number of occurrences for gene encoding PGP traits compared to NV soils (average count of genes: 35.7, 33.1 and 24.3, respectively). Functional genetic PGP potential of the 102 medium- and high-quality MAG confirmed the prevalence of multiple PGP traits in RS and RH (Supplementary Data S4).

### Microbial competition biomarkers are abundant in rhizosheath–root system compartments

BGCs encoding for secondary metabolite production, such as antibiotics, pigments and sunscreens [79], were analyzed (Fig. 5c and Supplementary Data S3). The vast majority of BGCs belonged to bacteria, especially *Actinobacteria*, with only 252 BGCs assigned to fungal guilds, possibly due to the low percentage of reads assigned to the latest (Supplementary Results S3). The bacterial BGCs significantly decreased in abundance from the RS to the NV soil metagenomes (527 ± 33 BGCs in RS, 269 ± 21 in RH and 37 ± 19 in NV; *p* < 0.0001; Fig. 5c), suggesting that the RS and RH microbiomes are subject to higher levels of inter-taxa competition than NV soil microbiomes. Beside their limited number, also the fungal BGCs become more prominent in proximity of the plant (5 fungal BGCs in NV, 103 in RH and 144 in RS). Many BGCs were found to encode for antibiotic production (RS = 36% ± 7%, RH = 28% ± 5% and NV = 9% ± 13%; Fig. 5c and Supplementary Data S3) but the RH and RS metagenomes were also significantly enriched in antibiotic resistance genes (*p* < 0.001). The number of phage-related contigs showed the same trend with 75 ± 15, 34 ± 16 and 2 ± 3 viral contigs in the RS, RH and NV soil metagenomes, respectively (Supplementary Result S3). Ninety three percent of these phage contigs were unclassified using protein-sharing networks. The classified viral contigs belonged to *Siphoviridae* (*Caudovirales*, dsDNA tailed phage; Supplementary Data S3), possibly infecting the dominant actinobacterial genera, such as *Streptomyces*, *Actinoplanes* and *Arthrobacter*. Similarly, we observed a significantly higher proportion of CRISPR-Cas systems, which are proxies for previous viral infection events and bacterial immunity acquisition processes [80], in the RS and RH metagenomes than in the NV soil metagenomes (*p* < 0.05; Supplementary Data S3).

## Discussion

In hyperarid desert ecosystems, resource (i.e., water and nutrients) availability is a key challenge for macro- and microorganisms. In this context, xerophytic plants and their root systems serve as important resource hotspots that affect both the structure and function of the surrounding edaphic microbial communities [15, 18, 81]. Here, we investigated the effects of the RS–root system of *S. ciliata* on the surrounding edaphic microbial community in the extremely oligotrophic gravel plain soils of the hyperarid central Namib Desert. *S. ciliata* is an amphiphytic speargrass which is abundant on rock-sandy gravel plains of the Namib Desert after spatio–temporally isolated rainfall events exceeding 20 mm [35]. A conspicuous adaptive trait evolved by *Stipagrostis* plants, and other xerophytic plants within the *Poaceae* family, is the development of a RS–root system [15], a cylindrical sheath of sand particles surrounding all elements of the root system. The RS is rich in exopolymers and mucigels produced by the root and its associated microorganisms [18, 22, 82] to efficiently retain water by reducing evapotranspiration [21, 83], as confirmed by the fact that the *S. ciliata* RS studied retained water more than a month after a light (<7 mm) rainfall.

An additional key adaptive trait of *S. ciliata* is the recruitment of specific microbial taxa and their functional capacity in the RS–root system. Indeed, the RS and RH edaphic niches have been described as “resource islands” and “diversity hotspots” in (hyper)oligotrophic and (hyper)arid soils [15, 18, 84, 85]. As already observed for Namib Desert dune speargrass species [15], *S. ciliata* recruits and assembles the microbial community in the RS–root system *via* strong deterministic niche-partitioning processes. While such communities have a reduced (RS) or similar (RH) compositional diversity than NV soil in term of richness, they present high functional redundancy (i.e., a same function can potentially be carried out by different microorganisms) with multiple taxa carrying a given PGP trait, including auxin and EPS production and provision of essential nutrients, e.g., P, N and Fe [15, 86–91]. Such functional redundancy suggests that the RS and RH compartments represent “functional shields” to support/ensure the fitness of the holobiont during extended dry periods, as well as “functional reservoirs” from which the plant selects its intimately associated endophytic communities.

RS and RH microbial communities of *S. ciliata* form more cohesive co-occurrence networks than those of NV soils, supporting the concept that both water and nutrient availability are determinant factors in the assembly and stability of desert edaphic microbial communities [37, 59, 92, 93]. Key components of the co-occurrence networks are microorganisms known to possess PGP traits that improve plant-fitness, such as Firmicutes of *Bacillus* and *Paenibacillus* genera. Members of these genera are often recognized as PGP taxa resistant/tolerant to drought [27, 72, 94–96] and have previously been found as connectors in RS-RH microbial networks of other Namib Desert speargrass species, namely *S. sabulicola*, *S. seelyae* and *Cladoraphis spinosa* [15]. This suggests that members of these bacterial genera have developed an intimate relationship, and possible co-evolution [97], with Namib Desert xerophytic plants forming RS–root structures. Similarly, the root fungal colonizers *Mortierella* sp., *Auxarthron* sp. and *Xylaria* sp. [98, 99] were also identified as important connectors in the RS and RH co-occurrence networks. These fungi support plant nutrient uptake [75, 100] and plant protection from herbivores and phytopathogens [101]. Their hyphae can further promote soil aggregation and stabilization, and therefore participate in the development of the RS structure surrounding plant roots [15], ultimately increasing soil water-holding capacity [102–105] and connectivity between edaphic microorganisms [90, 106, 107].

The inter-taxon interactions established between the microbial community members can either be positive (mutualism) or negative (competition) [15, 39, 90, 108]. While positive co-occurrences dominate in the oligotrophic NV soil networks, those negative increase along the RS and RH compartments (Table 2). This strongly suggests that desert edaphic microorganisms associated with the “limited niche-space” of the RS oasis compete more with each other than those inhabiting the unhospitable NV soil to reduce the invasion of possible competitors. The fact that a larger number of genes involved in antibiotic production, export and resistance was observed in the RS and RH strongly supports this view [93, 109]. As well, the presence of CRISPR-Cas proteins in these plant niches hints at defense mechanisms against the numerose and diverse edaphic phages [110] that might act as moderating agents within the community by controling population density [111].

**Fig. 6:**
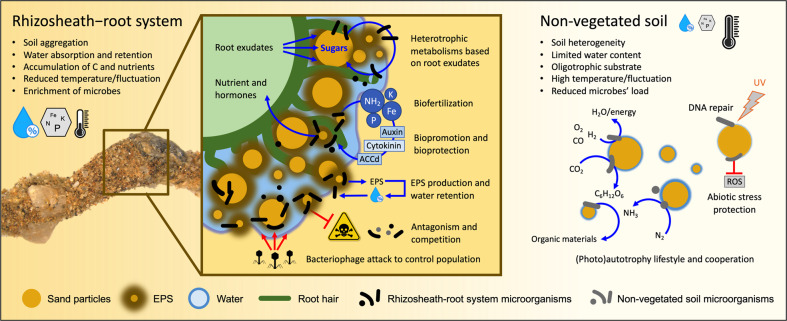
Ecological race and interactions of microbial partners attracted by the rich root–rhizosphere niche under limited available resources in the hyperarid desert. Schematic illustration of microbial communities associated with *S. ciliata* rhizosheath–root system (RS and RH) and NV soil and their contribute to niche-ecosystem functioning. Environmental, ecological, and functional characteristics of microbial communities associated with *S. ciliata* rhizosheath–root system and NV soil are reported considering both plant/soil–microbe and microbe–microbe interactions. Trophic modes and functions from metagenomes are resumed. More detailed explanation is reported in the “Discussion” section of the manuscript.

Altogether, our study supports the contention that plant RS–root systems represent resource islands and microbial density and competition hotspots. We therefore propose a dual selection process as a model of plant–microbe interaction in the colonization and occupation of the RS–root system niches (Fig. 6): to colonize the plant-associated niches desert edaphic microorganisms must (1) possess the capacity to improve the fitness and survival of the plant (plant selection) and be able (2) to compete successfully against other microorganisms (microbial competition); i.e., that both long-term plant–microbe co-evolution [112] and a microbial “arms race” [113, 114], respectively, co-occur. In contrast, NV soil desert microorganisms are less competitive but better equipped with self-sustaining processes to cope with the extreme/oligotrophic conditions to which they are exposed to. By some ecological analogies with the RS–root system, we speculate that similar processes should occur in other desert microbial refuge niches, such as lithic environments and biological soil crusts [8].

## Conclusions

The RS–root system of *S. ciliata* is an “oasis” for hyperarid desert edaphic microorganisms as it provides more favorable abiotic conditions (with more nutrients and water) in otherwise poly-extreme and highly oligotrophic gravel plains. Consequently, these refuge niches and resource islands are hotspots for which edaphic microorganisms compete intensively to colonize. This is particularly emphasized by the fact that, despite presenting similar diversity in term of species number, the RH communities presented a significantly higher microbial density than the NV soil communities. This is in agreement with our initial hypothesis that the RS–root system of *S. ciliata* acts as hotspot for microbial colonization. The microorganisms able to colonize and remain in association with the RS–root system are (1) functionally equipped to support the host (and their own) growth and survival through biopromotion and biofertilization activities, that is they have been selected for their plant growth promoting capabilities, and (2) they have been selected to compete against the other microorganisms (e.g., through antibiotic production and viral pathogen resistance). Indeed, while presenting more favorable abiotic conditions, the biotic pressure endured by the resource island microbial colonizers is also an important factor to be considered to disentangle the mechanisms regulating the colonization of such “resource islands” in poly-extreme deserts. By applying Darwin’s evolutionary theory [37], we further postulate that such density/competitive niches may also represent evolutionary hotspots that can enhance the resilience and success of the RS–root microbial communities and their host, i.e., the plant holobiont in the extremely harsh and inconsistent environmental conditions of deserts and those arising from climate change.

## Supplementary information

Supplementary Material

Supplementary Table S3

Supplementary Data S1

Supplementary Data S2

Supplementary Data S3

Supplementary Data S4

## Data Availability

The datasets generated during the current study are available in the NCBI SRA repository under the BioProject ID PRJNA628615. The raw, unassembled metagenomes are available in the NCBI SRA repository under the BioProject ID PRJNA731454.
