# Supplementary material for: The plant rhizosheath–root niche is an edaphic “mini-oasis” in hyperarid deserts with enhanced microbial competition
Source: ISME Commun. 2022 Jun 3;2:47. doi: 10.1038/s43705-022-00130-7 (PMC9723607; doi:10.1038/s43705-022-00130-7)
Supplement: Supplementary file 1 — Supplementary Material [file 43705_2022_130_MOESM1_ESM.pdf]

SUPPLEMENTARY MATERIALS of

The plant rhizosheath–root niche is an edaphic "mini-oasis" in hyperarid deserts with enhanced microbial competition

Ramona Marasco[1]*, Marco Fusi[1], Jean-Baptiste Ramond[2,3], Marc W. Van Goethem[1,2], Kholoud Seferji[1], Gillian Maggs-Kölling[4], Don A. Cowan[2] and Daniele Daffonchio[1]*

[1]King Abdullah University of Science and Technology (KAUST), Biological and Environmental Sciences and Engineering Division (BESE), Thuwal 23955-6900, Saudi Arabia
[2]University of Pretoria, Centre for Microbial Ecology and Genomics, Department of Biochemistry, Genetics and Microbiology, Pretoria, South Africa
[3]Department of Molecular Genetics and Microbiology, Pontificia Universidad Católica de Chile, Santiago, Chile
[4]Gobabeb — Namib Research Institute, Walvis Bay, Namibia

*Authors for correspondence: Ramona Marasco, e-mail: ramona.marasco@kaust.edu.sa; Daniele Daffonchio, e-mail: daniele.daffonchio@kaust.edu.sa

SUPPLEMENTARY METHOD

Supplementary Method S1. Metagenome-assembled genome (MAG) binning, dereplication, taxonomy and phylogeny. High-quality reads were mapped to their corresponding assembly using BBmap v38.94 [1] after assemblies were indexed as reference files. Mapped reads were converted using Samtools v1.8 [2]. Automatic binning was performed using MetaBAT2 v2.12.1 [3] and MaxBin 2.0 v2.2.7 [4]. DASTool v1.1.1 [5] was then used for dereplication with --search_engine diamond and has dependencies including Pullseq v1.0.2, Prodigal v2.6.3 [6], DIAMOND v0.9.14 [7] and BLASTP [8] to select for the best bins across binning algorithms. These dereplicated bins were then checked for completeness and contamination using CheckM v1.1.3 under the --lineage_wf pipeline [9]. Final MAG curation consisted of running MAGPurify v2.1.2 on all genomes to remove any incorrectly binned contigs [10]. MAGs were classified as high- or medium-quality according to current minimum information about a MAG (MIMAG) standard [11]. Genome taxonomies of all medium- and high-quality MAGs were initially determined with GTDB-Tk v1.0.2 [12] which relies on RefSeq release 89 (accessed 18-06-2019). Dependencies of GTDB-Tk include Prodigal v2.6.3 [6] and HMMER v3.1b2 [13] for gene identification, pplacer v1.1 [14] and FastTree 2 v1.2 [15] for placement of genomes into the reference tree, and FastANI v1.3 [16] to compute average nucleotide identity (ANI). We used iTOL v6.4 [17] for visualizing and refining the tree generated by GTDB-Tk. Prokka v1.14.6 [18] was used for genome annotations with the --kingdom option set as Bacteria or Archaea and the --metagenome argument implemented.

SUPPLEMENTARY RESULTS

Supplementary Result S1. Richness differences and replacement processes resulted in the assembly of relatively stable and low scattered microbial communities, with the only exception of endophytic bacterial communities that showed higher variability (high dispersion), possibly due to the low number of OTUs consistently present across the root tissue sampled (Supplementary Figure S6) and the different selection-forces depending on *S. ciliata* plant's status (*e.g.*, age and density). In its perennial form it is not possible to determine their age, which may affect microbe-interactions in plants [19].

Supplementary Result S2. The interactions (edges) among and between the three microbial groups studied (*i.e.*, bacteria-bacteria, fungi-fungi, bacteria-fungi, and the interactions including algae) were also niche-dependent. The RT and RS networks were particularly rich in interactions involving bacteria; the former being particularly dominated by bacteria-bacteria co-occurrences. In contrast, the RH and NV soil networks were dominated by interactions involving fungal OTUs (*i.e.*, fungi-fungi and fungi-bacteria). Algae-driven interactions increased from the RT to the NV soil, while bacteria-fungi ones decreased from the RS towards the NV soil. Analyses of nodes degree and betweenness revealed total of 289 bacterial and 292 fungal OTUs classified as hubs (grey boxes in Figure 4c), among which 87 and 57 were keystone species (red-dashed boxes in Figure 4c), respectively. 20 algal OTUs were defined as network hubs, none being keystone species. Results of hubs and keystone species are reported in the Supplementary Data S2.

Supplementary Result S3. Bacterial, fungal, and virus diversity from metagenomes. From metagenome data we confirmed the distribution pattern of bacteria and fungi across compartments obtained by meta-phylogenomic analysis amplicon-based (Figure 3). Analysis of ribosomal genes showed that all communities were dominated by OTUs belonging to Actinobacteria which are common soil colonists. Overall, five phyla were significantly enriched in the non-vegetated soil compared to the rhizosphere and rhizosheath, including *Chloroflexi* (ANOVA; corrected-$p < 0.00548$), *Deinococcota* (corrected-$p < 0.00739$), *Firmicutes* (corrected-$p < 0.00044$), *Gemmatimonadetes* (corrected-$p < 0.00843$), and *Omnitrophica* (corrected-$p < 0.01$). By contrast, *Alphaproteobacteria, Gammaproteobacteria* and *Patescibacteria* increased in relative abundance from non-vegetated soil to the rhizosphere and rhizosheath.

From the metagenome analysis, we did not recover many sequences belonging to fungi or eukaryotes generally using ribosomal marker genes. The same trend was observed when assigning taxonomy to the filtered reads using Kraken 2 [20]. In this instance we found that < 1% of all reads were of fungal origin. However, we did observe an increase in the number of sequences belonging to fungi from non-vegetated soil to the rhizosphere and rhizosheath in both ribosomal and raw read datasets. On average we found ~23,000 fungal sequences in the non-vegetated soil, ~66,000 in the rhizosphere and ~200,000 in the rhizosheath (Supplementary Data S3). Some of these fungal sequences were unassigned although ribosomal sequences were related to the thermotolerant fungus *Thielavia terrestris*, while the proportion of raw reads for *Aspergillus* and *Fusarium* became enriched in the rhizosphere and rhizosheath.

We also explored whether or not bacteriophages were common members of these soil communities. To do this we used VirSorter [21] of all assemblies to identify contigs that contained signatures of bacteriophages (capsid proteins, integrases, tail fiber proteins). We only retained phage contigs in categories 1, 2 (complete viral contigs) and 4, 5 (prophages) that were > 10 kb. These are uncultivated viral genomes (UViGs). Phages were then checked for quality using CheckV [22]. This showed that six phage genomes could be detected in non-vegetated soil communities, 101 in the rhizosphere assemblies and 223 in the rhizosheath assemblies.

SUPPLEMENTARY TABLES

Supplementary Table S1. Meteorological data from the Gobabeb weather station, which is part of the Southern African Science Service Centre for Climate Change and Adaptive Land Management (SASSCAL) network, Station ID: 8893 [23]. The station is located 9.8 km from the sampling site; April (in bold) is the month in which samples of *S. ciliata* rhizosheath root-system and non-vegetated soil have been collected. Data available at http://www.sasscalweathernet.org/weatherstat_monthly_we.php.

Month, 2017	Av. Air T (°C)	Min. Air T (°C)	Max. Air T (°C)	Rainfall (mm)	Humidity (%)
January	21.4	12.1	37.2	0	63.4
February	22.4	0	35.7	1.1	60
March	27.6	13.9	40.3	7.9*	42.6
**April**	**26.1**	**11.1**	**38.9**	**0**	**37.2**
May	26	11.9	36.5	0	25.2
June	20.4	6.1	34.2	0	37.8
July	19.1	4.3	34.1	0	37.9
August	19.6	5.1	35.4	0	31
September	17.1	5.8	39.6	0	65
October	17.9	6.4	34.5	0.3	56.8
November	21.2	9.4	39.9	2.5	50.7
December	21.4	10.2	36.2	0	56.5

* Days of rainfall: March, 17=0.1 mm and March, 18=7.8 mm

Supplementary Table S2. Analysis of the prokaryotic 16S rRNA gene and ITS region reads obtained from the sequencing. For each compartment are given the total number of reads discarded per category and the final number of reads further used in the analyses are reported for both bacterial (top) and fungal (bottom) communities.

**16S rRNA gene OTUs**	**Root tissue**		**Rhizosheath**		**Rhizosphere**		**Bulk soil**	
	**Reads**	**%**	**Reads**	**%**	**Reads**	**%**	**Reads**	**%**
Total pair-end reads (11935 OTUs)	883518		641328		658931		1380822	
Chloroplast/Mitochondria (44 OTUs)	341365	38.6	22172	3.5	638	0.1	10517	0.8
Archaea[#] (63 OTUs)	170	0.03	46	0.007	125	0.018	1076	0.08
Unassigned (2151 OTUs)	28177	3.2	2554	0.4	4497	0.7	25056	1.8
Low relative abundance (<0.01%; 8570 OTUs)	19728	2.2	47293	7.4	75451	11.5	214323	15.5
**Used for the analysis (1107 OTUs)**	**494078**	**55.9**	**569263**	**88.8**	**578220**	**87.8**	**1129850**	**81.8**

[#] Excluded from the analysis because none of the 63 archaeal OTUs showed relative abundances > 0.01%; data reported in the table as percentage represent the sum of relative abundance of the all the archaeal OTUs.

**ITS OTUs**	**Root tissue**		**Rhizosheath**		**Rhizosphere**		**Bulk soil**	
	**Reads**	**%**	**Reads**	**%**	**Reads**	**%**	**Reads**	**%**
Total pair-end reads (862 OTUs)	1808441		1547821		1588584		2325580	
Others, non-fungi (12 OTUs)	1176	0.1	5	0.0003	25		13695	0.6
Unassigned (311 OTUs)	1483	0.1	1071	0.1	1513	0.002	1190	0.1
Low relative abundance (<0.001%; 247 OTUs)	290	0.02	992	0.1	2787	0.1	609	0.03
***Trebuxia* used for the analysis (19 OTUs)**	**78**	**0.004**	**9**	**0.001**	**52**	**0.2**	**45267**	**1.9**
**Fungi used for the analysis (273 OTUs)**	**1805414**	**99.8**	**1545744**	**99.9**	**1584207**	**0.003**	**2264819**	**97.4**

Supplementary Table S3. List of genes analyzed from the metagenome dataset. Refer to Marasco et al 2021_Supplementary Table S3.xlxs

Supplementary Table S4. (**a**) Physico-chemical conditions of the gravel plain non-vegetated soil (NV), speargrasses rhizosphere (RH) and rhizosheath (RS); values of the three replicates are reported. (**b**) Characterization of non-vegetated soil structure reported as mean ± standard deviation (St.dev.) of three replicates.

a) Variable	Unit	Non-vegetated soil			Rhizosphere			Rhizosheath		
		NV-A	NV-B	NV-C	RH-A	RH-B	RH-C	RS-A	RS-B	RS-C
Water	%	0.263	0.230	0.222	0.195	0.256	0.207	0.443	0.411	0.437
pH	Unit	9.21	8.92	9.19	8.06	8.73	8.29	8.46	9.04	9.02
Conductivity	uS/cm	30.5	29.8	31.2	18	26.7	24.4	29.2	47	33.5
Salinity	ppt	0.016	0.016	0.016	0.009	0.014	0.013	0.015	0.024	0.017
Nitrite	µg/mg	0.00005	0.00006	0.00005	0.00006	0.00006	0.00006	0.00008	0.00009	0.00008
Nitrate	µg/mg	0.00272	0.00620	0.00197	0.00152	0.00181	0.00169	0.00154	0.00309	0.00195
Ammonium	µg/mg	0.00010	0.00018	0.00013	0.00101	0.00073	0.00399	0.00051	0.00127	0.00060
Phosphate	µg/mg	0.00255	0.00176	0.00191	0.00389	0.00344	0.00566	0.00514	0.01161	0.00557
Silicate	µg/mg	0.031	0.034	0.029	0.018	0.023	0.025	0.039	0.045	0.039
Ca	µg/mg	0.407	0.354	0.277	0.207	0.298	0.225	0.409	0.544	0.440
K	µg/mg	0.518	0.253	0.235	0.242	0.191	0.244	0.203	0.344	0.190
Mg	µg/mg	0.014	0.012	0.009	0.008	0.012	0.010	0.017	0.029	0.020
S	µg/mg	0.006	0.003	0.002	0.004	0.013	0.009	0.008	0.060	0.012

b) Structure	Unit	Average	St.dev.
Stones	> 5mm	16.26	1.97
Small stones	2-5 mm	2.85	0.30
Coarse sand	1-2 mm	6.11	1.90
Coarse sand	500-1000 µm	6.33	1.93
Medium sand	250-500 µm	10.64	0.57
Fine sand	100-250 µm	28.64	1.01
Very fine sand	53-100 µm	23.89	4.22
Clay	<53 µm	2.18	1.49
Silt	<53 µm	3.10	0.56

Supplementary Table S5. Results of the *adonis* permutational multivariate analysis of variance. Partitioning squares were calculated using the Bray-Curtis similarity distance matrices of bacteria and fungi.

Factor	Bacteria - R^2	Fungi - R^2
**Compartment**	**0.62924**	**0.62814**
Residual	0.37076	0.37186

Supplementary Table S6. Results of the multivariate GLM analysis (pair-wise tests) are reported for bacterial and fungal communities associated with the rhizosheath-root system compartments and non-vegetated soil. The *p*-values indicated in bold are significant.

	Bacteria		Fungi	
**Pairwise comparison**	**$Dev_{1,8}$**	***p***	**$Dev_{1,8}$**	***p***
Root tissues vs Rhizosheath	11641	**0.002**	1062	**0.003**
Root tissues vs Rhizosphere	14054	**0.005**	1051	**0.009**
Root tissues vs Non-vegetated soil	15372	**0.002**	2225	**0.002**
Rhizosheath vs Rhizosphere	3654	**0.014**	547	0.073
Rhizosheath vs Non-vegetated soil	13314	**0.002**	2436	**0.006**
Rhizosphere vs Non-vegetated soil	11137	**0.004**	1514	**0.001**

Supplementary Table S7. Richness (number of OTUs) and evenness values are reported as mean ± standard deviation (st.dev.) of ten replicates for each rhizosheath-root system compartment and non-vegetated soil. Different lower-case letters indicate the significatively of the multiple comparison Tukey's test, $p < 0.05$.

**Compartment**	**Bacterial richness**			**Bacterial evenness**		
	**Mean**	**St.dev.**	**Tukey's test**	**Mean**	**St.dev.**	**Tukey's test**
Root tissue	57	27	a	0.498	0.186	a
Rhizosheath	769	64	b	0.679	0.051	b
Rhizosphere	899	36	c	0.759	0.027	bc
Non-vegetated soil	893	40	c	0.841	0.013	c

**Compartment**	**Fungal richness**			**Fungal evenness**		
	**Mean**	**St.dev.**	**Tukey's test**	**Mean**	**St.dev.**	**Tukey's test**
Root tissue	46	9	a	0.445	0.057	a
Rhizosheath	113	8	b	0.483	0.077	a
Rhizosphere	117	16	bc	0.532	0.091	ab
Non-vegetated soil	138	33	c	0.616	0.056	b

Supplementary Table S8. Relative abundance of taxa/classes detected in bacterial and fungal communities expressed as mean percentage ± standard deviation. Results of Kruskal-Wallis tests are reported (Kruskal-Wallis statistic and *p*-value); results of Dunn's multiple comparisons tests among compartments are indicated by lower case letters (different letters, $p < 0.05$).

**Bacterial phylum/class**	**KW test**	**Root tissue**	**Rhizosheath**	**Rhizosphere**	**Non-vegetated soil**
Actinobacteria	15.5, *p*=0.0014	15±20 a	44±10 b	41±9 ab	46±7 b
Alphaproteobacteria	24.4, *p*<0.0001	11±29 a	27±6 c	26±4 bc	15±3 ab
Firmicutes	22.5, *p*=0.0007	59±33 a	3±2 b	4±1 b	4±4 b
Bacteroidetes	25.9, *p*<0.0001	1±2 a	9±2 b	9±2 b	6±3 ab
Gammaproteobacteria	1.43, *p*>0.05	14±20	7±3	7±3	3±1
Chloroflexi	36.4, *p*<0.0001	0 a	0.8±0.4 ab	2±1 bc	12±4 c
Acidobacteria	35.9, *p*<0.0001	0.1±0.3 a	0.9±0.3 ab	2±0.7 bc	6±1 c
Patescibacteria	33.5, *p*<0.0001	0 a	4±2 b	3±1 b	0.7±0.3 a
Verrucomicrobia	22.4, *p*<0.0001	0.1±0.2 a	2±0.6 b	2±1 b	2±1 b
Deltaproteobacteria	26.7, *p*<0.0001	0.1±0.2 a	1±0.5 b	2±1 b	1±0.2 b
Others (< 1%)	34.1, *p*<0.0001	0 a	1±0.4 ab	2±1 bc	5±2 c

**Fungal class**	**KW test**	**Root tissue**	**Rhizosheath**	**Rhizosphere**	**Non-vegetated soil**
Sordariomycetes	23.9, *p*<0.0001	63±22 a	65±23 a	50±12 a	12±2 b
Dothideomycetes	26.3, *p*<0.0001	4±3 a	3±2 a	21±20 ab	55±15 b
Eurotiomycetes	6.3, *p*=0.098	9±15	10±9	14±10	8±9
Agaricomycetes	8.1, *p*=0.055	9±15	18±24	7±10	1±1
Pezizomycetes	30.3, *p*<0.0001	<0.01 a	0.1±0.1 b	2±5 b	6±6 c
Tremellomycetes	30.1, *p*<0.0001	0.01±0.05 a	0.03±0.02 ab	0.5±0.6 bc	3.4±3.8 c
Rhizophylyctidomycetes	34.1, *p*<0.0001	0 a	0 a	0.2±0.6 a	5.5±2.8 b
Wallemiomycetes	9.9, *p*=0.019	<0.01 a	0.05±0.09 ab	0.4±1.2 ab	1.2±2.4 b
Trebouxiophyceae	29.5, *p*<0.0001	<0.01 a	0 a	<0.01 a	1.8±1.3 b
Unclassified Fungi	17.4, *p*=0.0006	14±12 a	2±3 b	3±6 b	3±1 b
Others (< 1%)	22.2, *p*<0.0001	0.2±0.3 a	0.1±0.09 a	0.5±0.6 ab	2±1.5 b

SUPPLEMENTARY FIGURES

Supplementary Figure S1. Pictures showing (**a**) *Stipagrostis cilata* as dominant plant species in gravel plan forming a monospecific vegetation cover, (**b**) the sampling site (arrows indicate some of the *S. ciliata* clumps) and (**c**) an example of a live *S. ciliata* clump (identified by the growth of new green leaves; yellow strip = 10 cm long) collected during the sampling. (**d**) Visualization under a stereomicroscope of an intact rhizosheath-root portion showing associated minerals, stones and sand particles. Bar, 1 mm. (**e**–**g**) Examples of stones attached/embedded to/into the rhizosheath structure. Possible fungal hyphae and root hairs strongly attached to the stones are visible (black arrows), along with signs of biological rock weathering mediated by microorganisms and/or root action. Bars, 0.5, 1, and 0.2 mm in panels **e**, **f,** and **g**, respectively.

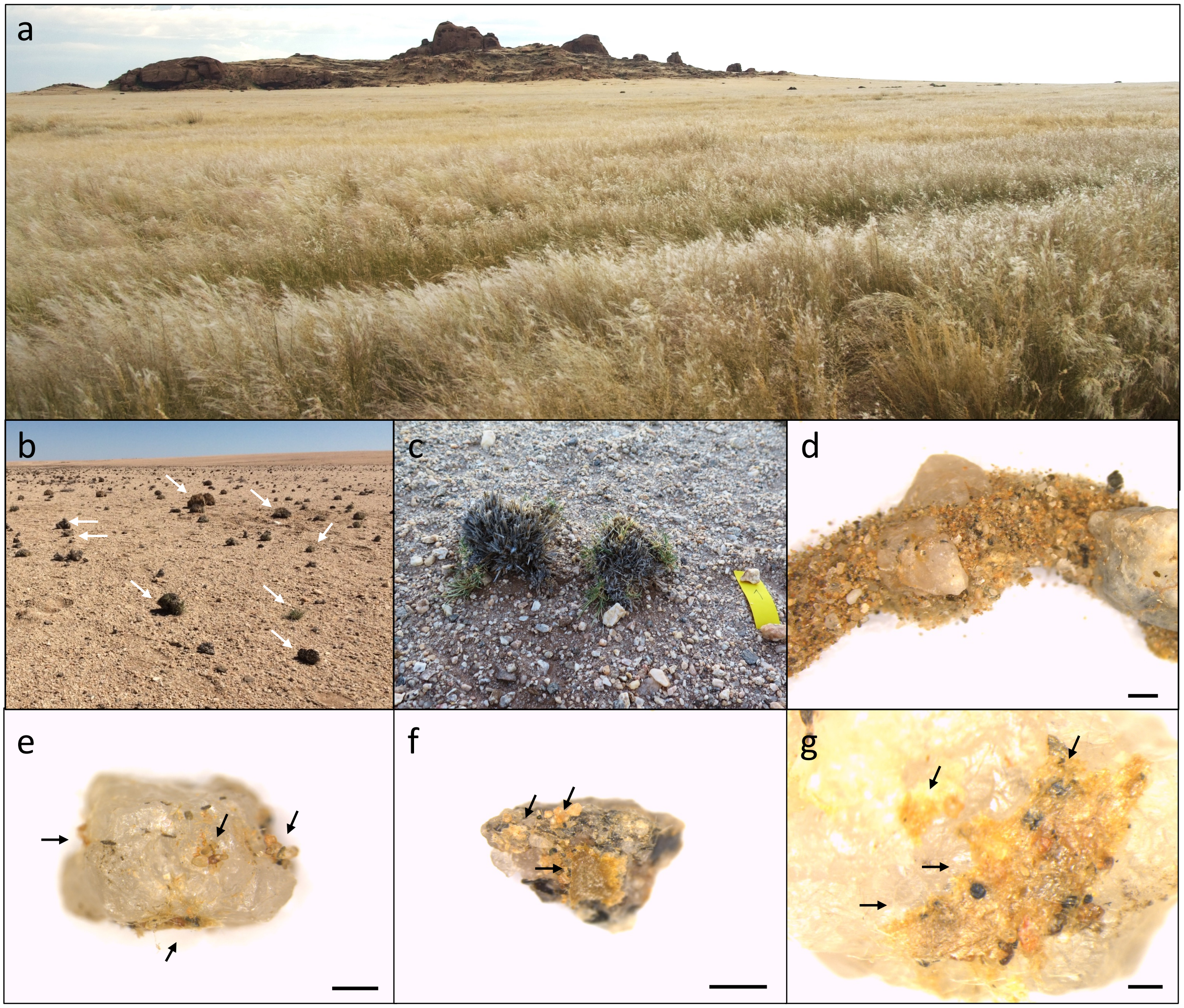

Supplementary Figure S2. Rarefaction curves for the bacterial (upper part) and fungal (lower part) sequencing datasets. For each compartment (root tissues, rhizosheath, rhizosphere and bulk soil) the rarefaction curves of the ten samples sequenced were reported. The rarefaction curves for all samples tended to and, in most cases, reached the plateau. This demonstrated that their sequencing depths captured was important and therefore all samples were included in the analyses.

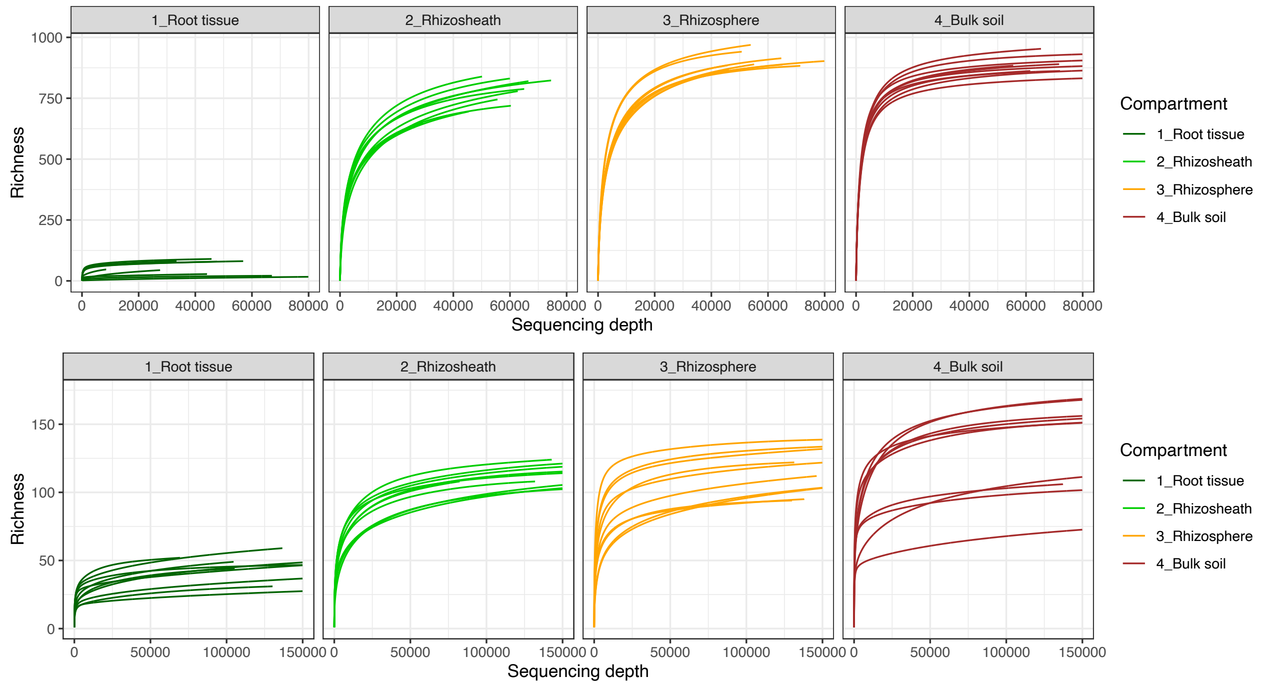

Supplementary Figure S3. Plots of residuals evaluating the quality of our model assumptions (negative_binomial distribution) for the analysis of (**a**) bacterial and (**b**) fungal communities. Both plots show random scatter of points.

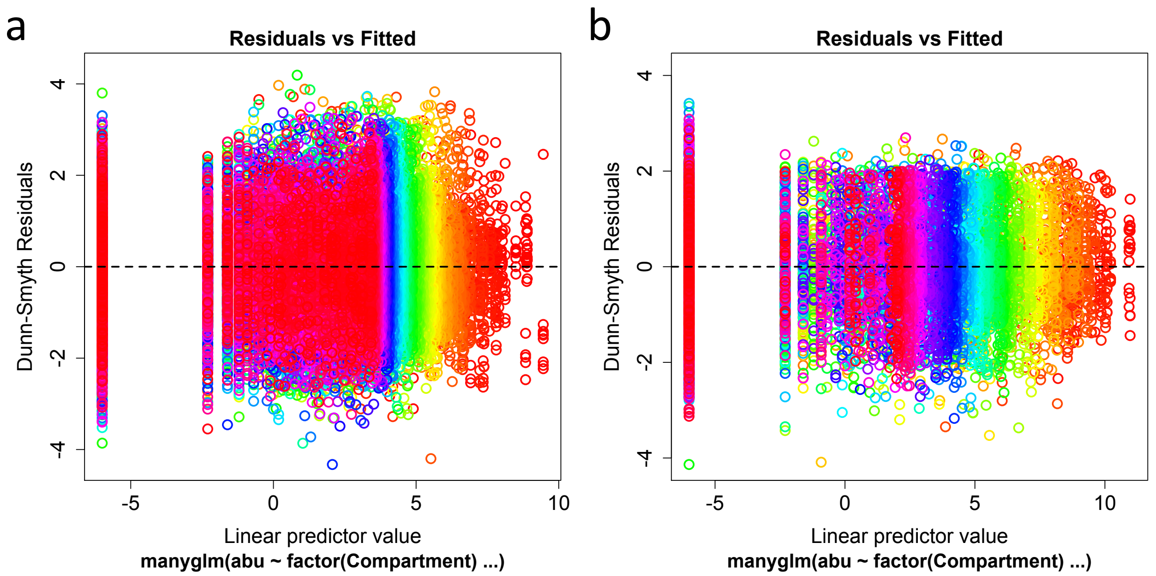

Supplementary Figure S4. Read redundancy of the metagenomes. The rhizosheath (green) metagenomes had the highest read redundancy, followed by the rhizosphere metagenomes (blue) and non-vegetated soil metagenomes (brown).

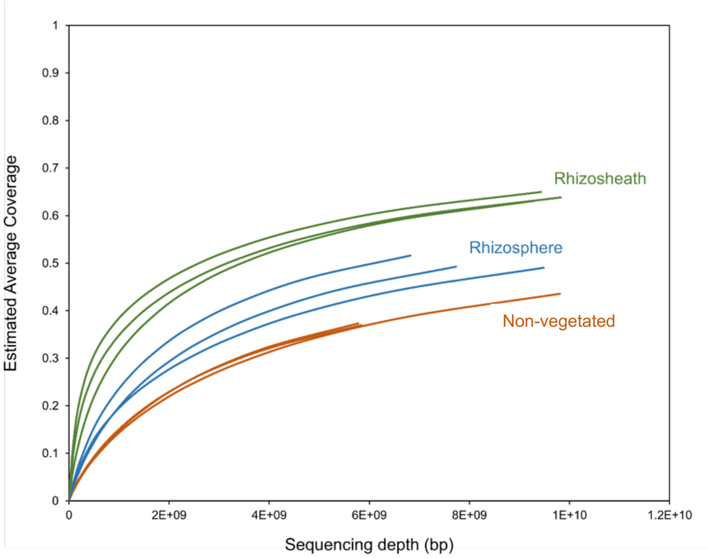

Supplementary Figure S5. Venn diagram depicting bacterial and fungal OTUs distribution. Bigger numbers indicate the relative abundance of OTUs expressed as percentage, while numbers in brackets are the percentage of these OTUs on the total number of OTUs (*i.e.*, 1701 and 292 OTUs for bacteria and fungi, respectively). Number in bold indicate the relative abundance such OTUs associated with the rhizosheath-root system compartments (*i.e.*, root tissues, rhizosphere, and rhizosheath) that originated from non-vegetated soil.

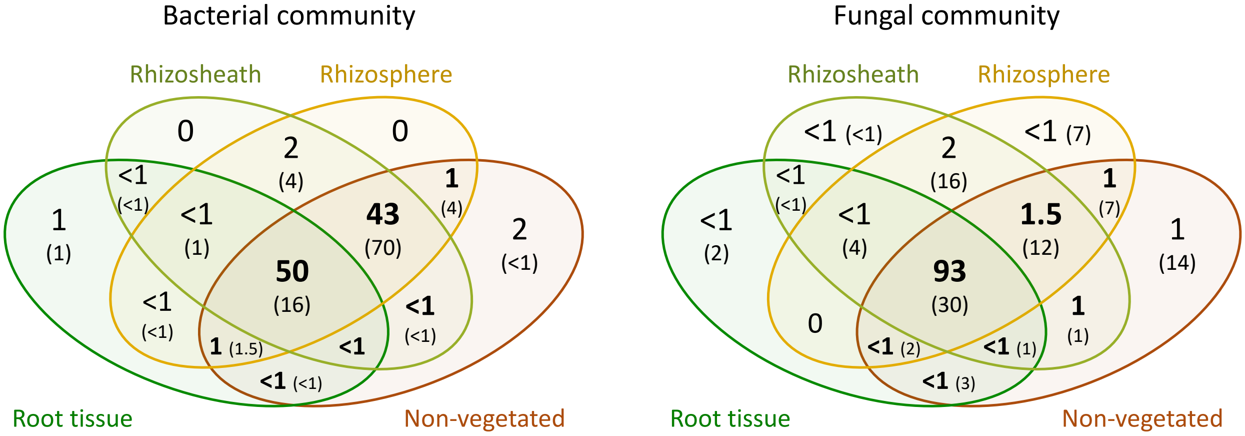

Supplementary Figure S6. Rank of sample and OTUs frequency for bacteria (upper part) and fungi (lower part) along the rhizosheath-root system compartments and non-vegetated soil. It is represented as relationship between OTUs prevalence in speargrasses root tissues, rhizosheath, rhizosphere, and non-vegetated soil compartments (samples degree, $n_{min} = 1$ and $n_{max} = 10$) and abundance (measured by the number of OTUs' reads in each compartment) for bacterial and fungal OTUs.

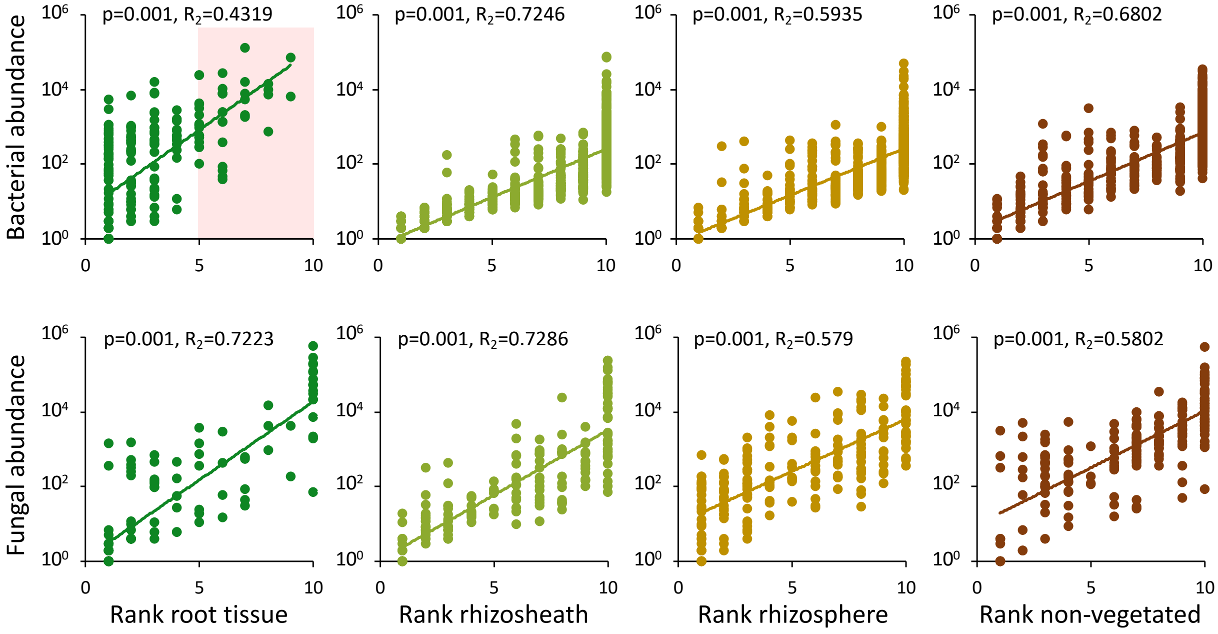

Supplementary Figure S7. Node (**a**) connectedness (normalized-degree) and (**c**) centrality (betweenness) of microbial co-occurrence networks of the rhizosheath-root system compartments (RT: root tissue; RS: rhizosheath; RH: rhizosphere) and non-vegetated soil (NV). Dots represent single observations and the lines among the dot clouds represent medians. Among compartments (RT, RS, RH and NV), set of dots with the same lower-case letters are not statistically different. Node (**b**) connectedness and (**d**) centrality are visualized here separating three microbial components (*i.e.*, B: bacteria, F: fungi, and A: algae). Lower-case letters indicate differences among the microbial components in each compartment. Centrality did not show significant difference among compartments and among microbial components in compartment networks.

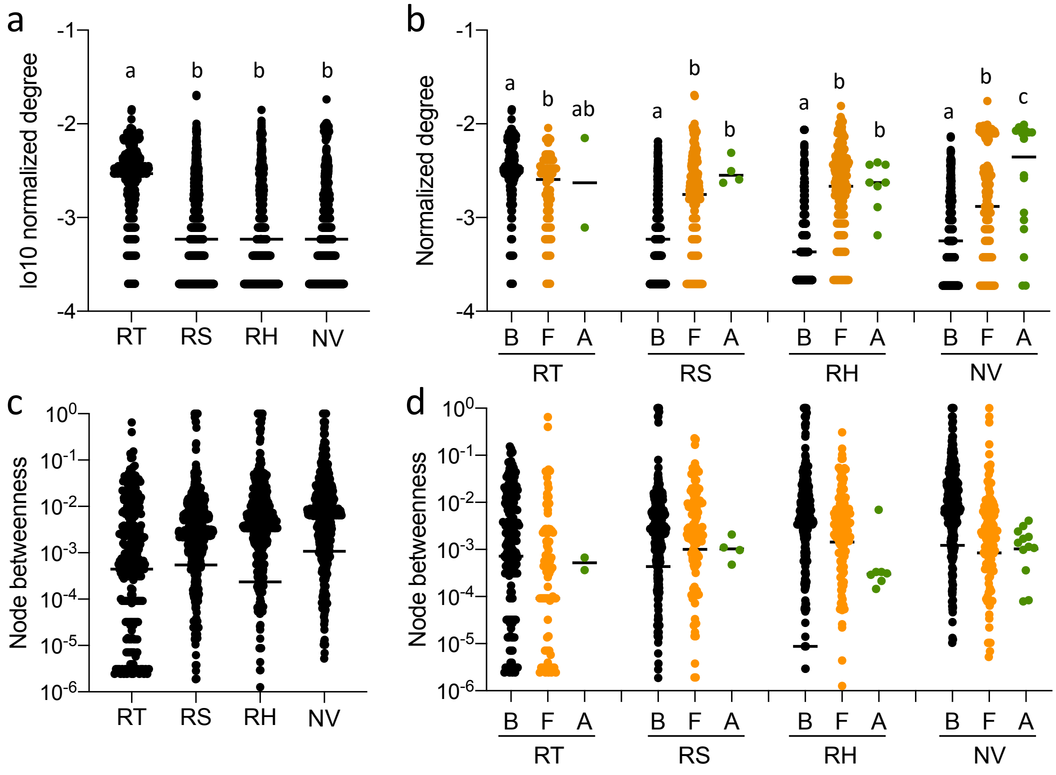

Supplementary Figure S8. Prokaryotic and fungal community composition based on ribosomal proteins obtained from metagenome analysis. The results show similar taxonomic patterns to the one obtained by the amplicon sequencing. Note that root tissues were not included in the metagenome analysis.

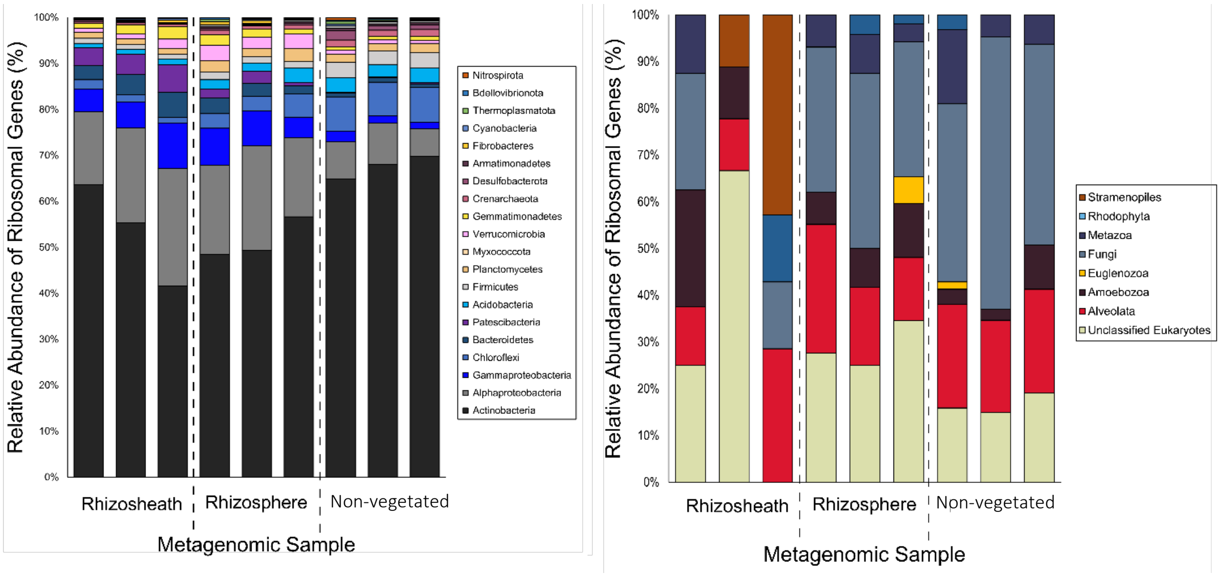

SUPPLEMENTARY DATA

Supplementary Data S1. Bacterial and fungal taxonomy across the different compartments analyzed; RT: root tissue, RS: rhizosheath, RH: rhizosphere and NV: non-vegetated soil.

Supplementary Data S2. Hubs and keystone species' taxonomic affiliations in the co-occurrence networks of root tissue (RT), rhizosheath (RS), rhizosphere (RH) and non-vegetated soil (NV).

Supplementary Data S3. Analysis of metagenome data; (1) raw metagenome statistics, (2) metagenome assembly statistics, (3) genes differentially enriched between rhizosheath, rhizosphere and non-vegetated soil, (4) biosynthetic gene clusters (BGCs) detected across rhizosheath, rhizosphere and non-vegetated soil, and (5) Viral contigs detected in assembled metagenomes are reported.

Supplementary Data S4. Metagenome-assembled genome (MAG) and associated beneficial PGP traits.

SUPPLEMENTARY REFERENCES

1. Bushnell B. BBMap: a fast, accurate, splice-aware aligner. 2014. Berkeley, CA (United States).
2. Li H, Handsaker B, Wysoker A, Fennell T, Ruan J, Homer N, et al. The sequence alignment/map format and SAMtools. *Bioinformatics* 2009; **25**: 2078–2079.
3. Kang DD, Li F, Kirton E, Thomas A, Egan R, An H, et al. MetaBAT 2: an adaptive binning algorithm for robust and efficient genome reconstruction from metagenome assemblies. *PeerJ* 2019; **7**: e7359.
4. Wu Y-W, Simmons BA, Singer SW. MaxBin 2.0: an automated binning algorithm to recover genomes from multiple metagenomic datasets. *Bioinformatics* 2016; **32**: 605–607.
5. Sieber CMK, Probst AJ, Sharrar A, Thomas BC, Hess M, Tringe SG, et al. Recovery of genomes from metagenomes via a dereplication, aggregation and scoring strategy. *Nat Microbiol* 2018; **3**: 836–843.
6. Hyatt D, Chen G-L, LoCascio PF, Land ML, Larimer FW, Hauser LJ. Prodigal: prokaryotic gene recognition and translation initiation site identification. *BMC Bioinformatics* 2010; **11**: 119.
7. Buchfink B, Reuter K, Drost H-G. Sensitive protein alignments at tree-of-life scale using DIAMOND. *Nat Methods* 2021; **18**: 366–368.
8. Altschul SF, Gish W, Miller W, Myers EW, Lipman DJ. Basic local alignment search tool. *J Mol Biol* 1990; **215**: 403–410.
9. Parks DH, Imelfort M, Skennerton CT, Hugenholtz P, Tyson GW. CheckM: assessing the quality of microbial genomes recovered from isolates, single cells, and metagenomes. *Genome Res* 2015; **25**: 1043–1055.
10. Nayfach S, Shi ZJ, Seshadri R, Pollard KS, Kyrpides NC. New insights from uncultivated genomes of the global human gut microbiome. *Nature* 2019; **568**: 505–510.
11. Bowers RM, Kyrpides NC, Stepanauskas R, Harmon-Smith M, Doud D, Reddy TBK, et al. Minimum information about a single amplified genome (MISAG) and a metagenome-assembled genome (MIMAG) of bacteria and archaea. *Nat Biotechnol* 2017; **35**: 725–731.
12. Chaumeil P-A, Mussig AJ, Hugenholtz P, Parks DH. GTDB-Tk: a toolkit to classify genomes with the Genome Taxonomy Database. *Bioinformatics* 2019; **36**: 1925–1927.
13. Eddy SR. Accelerated profile HMM searches. *PLoS Comput Biol* 2011; **7**: e1002195.
14. Matsen FA, Kodner RB, Armbrust EV. pplacer: linear time maximum-likelihood and Bayesian phylogenetic placement of sequences onto a fixed reference tree. *BMC Bioinformatics* 2010; **11**: 1–16.
15. Price MN, Dehal PS, Arkin AP. FastTree 2 – Approximately maximum-likelihood trees for large alignments. *PLoS One* 2010; **5**: e9490.
16. Jain C, Rodriguez-R LM, Phillippy AM, Konstantinidis KT, Aluru S. High throughput ANI analysis of 90K prokaryotic genomes reveals clear species boundaries. *Nat Commun* 2018; **9**: 1–8.
17. Letunic I, Bork P. Interactive Tree Of Life (iTOL) v5: an online tool for phylogenetic tree display and annotation. *Nucleic Acids Res* 2021; **49**: W293–W296.
18. Seemann T. Prokka: rapid prokaryotic genome annotation. *Bioinformatics* 2014; **30**: 2068–2069.
19. Wagner MR, Lundberg DS, del Rio TG, Tringe SG, Dangl JL, Mitchell-Olds T. Host

genotype and age shape the leaf and root microbiomes of a wild perennial plant. *Nat Commun* 2016; **7**: 12151.

20. Wood DE, Lu J, Langmead B. Improved metagenomic analysis with Kraken 2. *Genome Biol* 2019; **20**: 257.
21. Roux S, Enault F, Hurwitz BL, Sullivan MB. VirSorter: mining viral signal from microbial genomic data. *PeerJ* 2015; **3**: e985.
22. Nayfach S, Camargo AP, Schulz F, Eloe-Fadrosh E, Roux S, Kyrpides NC. CheckV assesses the quality and completeness of metagenome-assembled viral genomes. *Nat Biotechnol* 2021; **39**: 578–585.
23. Kaspar F, Helmschrot J, Mhanda A, Butale M, Clercq W De, Kanyanga JK, et al. The SASSCAL contribution to climate observation, climate data management and data rescue in Southern Africa. *Adv Sci Res* 2015; **12**: 171–177.
